# Senescent Fibroblasts Impair B Cell Proliferation via Cell–Cell Contact

**DOI:** 10.1111/acel.70766

**Published:** 2026-10-16

**Authors:** Kassandra Vezyrgianni, Elizabeth MacLeod, Abigail Nutley, Yuchen Zhu, Alice E. Denton

**Affiliations:** ^1^ Department of Immunology and Inflammation Imperial College London London UK

**Keywords:** B cells, fibroblasts, senescence, vaccines

## Abstract

Ageing profoundly impairs vaccine efficacy, leaving older individuals highly susceptible to infectious diseases despite vaccination. Although B cells retain the intrinsic capacity to generate germinal centre (GC) responses in older people, age‐associated defects in T cells, dendritic cells and follicular dendritic cells (FDCs) limit the development of high‐affinity, class‐switched antibody responses. Here, we hypothesised that senescent fibroblasts disrupt B cell activation and expansion, thus contributing to the diminution of the humoral response in ageing. To test this hypothesis, we established an in vitro system in which human tonsil‐derived YK6 fibroblasts expressing CD40L are co‐cultured with PBMC‐derived B cells. B cell activation, proliferation, and phenotype in response to low‐ and high‐stimulation conditions was determined in co‐culture with control or bleomycin‐treated YK6 fibroblasts. Control YK6 fibroblasts promoted B‐cell activation and proliferation in both 2D and 3D cultures, whereas bleomycin‐treated fibroblasts robustly inhibited B‐cell proliferation without impairing early activation. Conditioned media from bleomycin‐treated fibroblasts partially reduced B cell proliferation, although to a lesser extent than direct co‐culture, suggesting cell–cell contact was key to inhibiting B cell proliferation. Bleomycin‐treated YK6 fibroblasts upregulated genes associated with impaired lymphocyte responses, such as *PDCD1LG2*, which may impact B cell activation. Indeed, B cells co‐cultured with bleomycin‐treated fibroblasts failed to fully initiate glycolysis and were essentially unable to synthesise new protein. Together, these findings suggest that senescence‐like changes in fibroblasts could restrict B‐cell proliferative capacity, which may contribute to impaired B cell responses in ageing.

## Graphical abstract

Tonsil‐derived fibroblasts support B cell activation and proliferation in response to stimulation in ex vivo 3D co‐culture. However, when rendered senescent using bleomycin, tonsil‐derived fibroblasts impair B cell responses. The B cells acquire activation markers and partially activate glycolysis, but are unable to proliferate or synthesise new proteins.

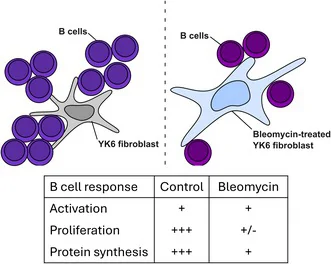

## 1 Introduction

Vaccination is an excellent public health measure that limits debilitating infections through the production of pathogen‐specific antibodies that limit (re)infection. Vaccines can be highly effective, inducing strong responses that protect individuals from infections and severe disease outcomes; however, there are many at‐risk populations for whom current vaccines fall short, such as older individuals (Gavazzi and Krause 2002). Ageing is a major barrier to effective vaccine‐elicited immunity, and this has a double impact on older people, who not only experience increased morbidity and mortality from infections but are also left less protected from current vaccine strategies, including the more novel mRNA vaccines (Allen et al. 2020; Palacios‐Pedrero et al. 2022).

Protective high affinity class‐switched antibodies are secreted by plasma cells, which are themselves derived from a specialised immune reaction, termed the germinal centre (GC) response. The GC response develops in a stepwise manner: B cells interact with T cells prior to GC formation through CD40‐CD40L interaction; following a positive interaction, T and B cells differentiate into GC T and B cells and move into the B cell follicle to form the GC. Once the GC is established, an iterative process of somatic hypermutation and T cell‐mediated selection drives affinity maturation of B cells (Crotty 2019; De Silva and Klein 2015; Hirth et al. 2019). Follicular dendritic cells (FDCs), a specialised type of fibroblast, coordinate the movement and localisation of T and B cells within the GC and also act as an antigen library to support the ongoing GC response (Heesters et al. 2014; Martínez‐Riaño et al. 2023). The ultimate output of the GC is plasma cells and memory B cells that can produce high affinity class switched antibodies specific for the vaccine antigen(s) that mediate the protective response.

Aged B cells are largely capable of contributing to GC responses (Denton et al. 2022; Yang et al. 1996) while defects in both T cells (Eaton et al. 2004; Lefebvre et al. 2012, 2016) and the microenvironment (Lancaster 2023; Turner and Mabbott 2017) contribute to the diminution of the GC response in ageing. We recently showed using heterochronic parabiosis that age‐associated defects in the microenvironment are a major driver of reduced humoral responses in ageing. Ageing impaired the activation and expansion of FDCs, which was associated with reduced GC formation despite the presence of young T and B cells within the lymph node (Denton et al. 2022). Other studies have demonstrated that ageing negatively impacts FDC functions, compromising expression of CXCL13, a B cell‐attracting chemokine (Minges Wols et al. 2010), and adhesion molecules and Fc and complement receptors (Aydar et al. 2002, 2003) that are essential for communication with, and antigen display to, B cells. Together these studies point to an age‐associated FDC‐intrinsic defect that impacts on B cell responses, although the drivers of FDC dysfunction in ageing have not been defined. Expression of p21 is increased in lymphoid fibroblasts isolated from 18 to 20 month old mice (Deng et al. 2025), indicating that lymphoid fibroblasts may become senescent in ageing. To test whether senescent fibroblasts contribute to the impaired B cell response observed in ageing we developed a model in which human tonsil‐derived YK6 fibroblasts (Caeser et al. 2019) were rendered senescent prior to co‐culture with B cells. We found that B cells were unable to proliferate in the presence of bleomycin‐treated YK6 fibroblasts (Bleo‐YK6), despite evidence of undergoing activation. Conditioned media (CM) from Bleo‐YK6 partially inhibited B cell proliferation, though to a lesser extent than co‐culture with Bleo‐YK6 fibroblasts, suggesting that surface‐expressed proteins are the major contributor to impaired B cell proliferation in this model. Mechanistically, co‐culture of B cells with bleomycin‐treated fibroblasts led to impaired upregulation of glucose importers and an inability to synthesise proteins, suggesting a failure to become ‘fully’ activated. Our study suggests that fibroblast‐intrinsic age‐associated defects may directly impact B cell responses and thus impair the GC response in ageing.

## 2 Results

### 2.1 Fibroblasts Promote B Cell Activation and Proliferation In Vitro

To investigate the impact of fibroblast senescence on B cell activation, we first established an in vitro model of low‐ and high‐stimulation of PBMC‐derived naïve‐enriched B cells. Naïve‐enriched CellTrace Violet (CTV)‐labelled B cells were stimulated with anti‐IgM, CpG dinucleotides and soluble CD40L for low stimulation, with the addition of interleukin (IL)‐2, IL‐4 and IL‐6 for high stimulation. We collected B cells two, three, four and seven days after stimulation; live, CTV+CD19+ B cells (Figure S1A) were analysed by flow cytometry. In the absence of stimulation, we saw no B cell proliferation over the seven‐day time course (Figure 1A). For both low‐ and high‐stimulation conditions, B cells began dividing at day three, with 40% and 60% of B cells proliferating in low‐ and high‐stimulation conditions, respectively, with B cell proliferation progressing faster in high‐stimulation conditions (Figure 1B,C). We also measured B cell activation by expression of CD69. In the absence of stimulation, we saw partial upregulation of CD69 at day seven (Figure 1D). At day two, 80% of B cells in low‐ and high‐stimulation conditions were CD69+ (Figure 1E,F). In low‐stimulation conditions, the proportion of CD69+ cells gradually decreased by day seven (Figure 1E), while CD69 expression was more variable in high‐stimulation B cells at the end of the experiment (Figure 1F).

**FIGURE 1:**
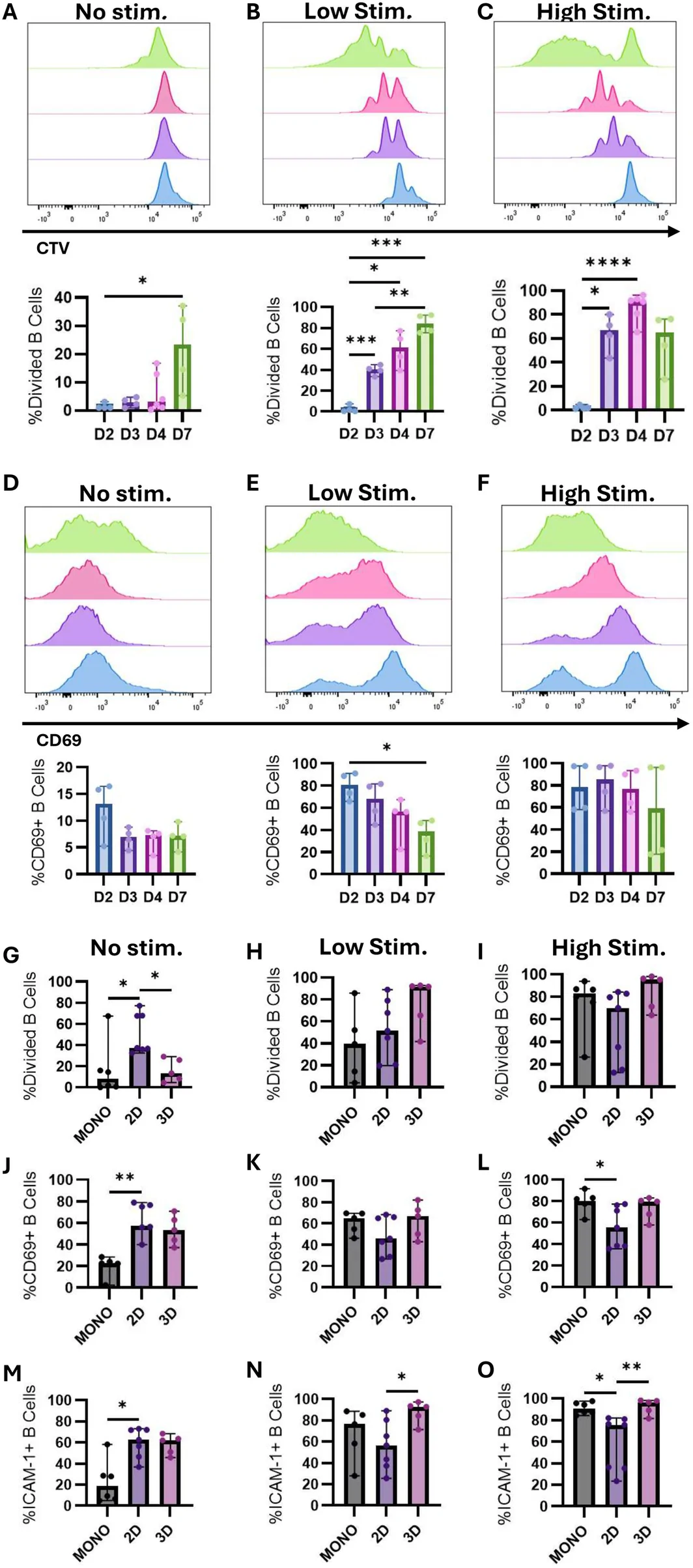
Co‐culture of stimulated B cells with YK6 fibroblasts can enhance early B cell activation. Naïve‐enriched PBMC‐derived B cells were labelled with CTV, cultured for two, three, four or seven days in no‐, low‐ and high‐stimulation conditions, and analysed by flow cytometry. (A–C) Representative flow cytometry histograms (top) and the proportion of divided B cells (bottom) for no‐ (A), low‐ (B) and high‐ (C) stimulation conditions. (D–F) Representative flow cytometry histograms (top) and the proportion of CD69+ B cells for no‐ (D), low‐ (E) and high‐ (F) stimulation conditions. (G–O) Naïve‐enriched PBMC‐derived B cells were labelled with CTV, cultured alone (mono) or co‐cultured with YK6 fibroblasts in 2D (plates) or 3D (Matrigel) for four days, then analysed by flow cytometry. (G–I) The proportion of divided B cells in low‐ (G), low‐ (H) and high‐ (I) stimulation conditions. (J–O) Bar graphs showing the proportion of CD69+ (J–L) and ICAM‐1+ (M–O) B cells for no‐ (J, M), low‐ (K, N) and high‐ (L, O) stimulation conditions. Gating strategy shown in Figure S1A. Data are pooled from seven independent experiments representing eleven PBMC donors. Statistical significance was determined with Kruskal‐Wallis test with Dunn's multiple comparisons, *p < 0.05, **p < 0.01, ***p < 0.001, ****p < 0.0001.

To understand whether fibroblasts augment B cell activation we employed YK6 fibroblasts, a human tonsil‐derived fibroblast line that has been immortalised via overexpression of *TERT*, *TP53* and *CDK4* and engineered to express CD40L and BAFF (Caeser et al. 2021), herein called YK6 fibroblasts. YK6 fibroblasts are derived from HK cells, an FDC‐like cell line (Kim et al. 1994). To determine if YK6 fibroblasts resemble human FDCs we tested the expression of key FDC genes based on flow cytometry and single cell RNAseq data from Heesters et al. (2021). Our flow cytometry data show that YK6 fibroblasts lack key FDC markers (CR1, LICAM, CD32b and CD23), although they did express CD73 and PD‐L2 (Figure S2A). We further assessed the transcriptome of YK6 fibroblasts and found that they more closely resembled the B zone‐supportive fibroblasts, FDCs and marginal reticular cells (MRCs), than T‐zone fibroblastic reticular cells (FRCs) (Figure S2B). Thus, cultured YK6 fibroblasts share features with human lymph node fibroblasts that interact with B cells in vivo.

To determine the impact of fibroblasts on B cell activation, we co‐cultured PBMC‐derived naïve‐enriched B cells with YK6 fibroblasts in no‐, low‐ and high‐stimulation conditions. B cell responses were analysed four days after stimulation. Co‐culture with YK6 fibroblasts with no additional stimulation induced B cell proliferation and activation, as measured by CTV dilution and upregulation of CD69 and ICAM‐1 expression (Figure 1G,J,M). The B cell response to low‐ and high‐stimulation conditions was not different between monocultured B cells and B cells co‐cultured with YK6 cells in standard 2D conditions (Figure 1H,I,K,L,N,O), except for the high stimulation conditions where a lower proportion of CD69+ (Figure 1L) and ICAM‐1+ (Figure 1O) B cells was observed. We hypothesised that the reduced B cell activation may be due to rapid proliferation of YK6 fibroblasts, which comprised up to 10% of the cells in culture in 2D conditions after four days. Thus, we conducted these experiments in a 3D setting using Matrigel. We observed a subtle increase in proliferation (Figure 1H,I) and CD69 expression (Figure 1K,L), and a significant increase in the proportion of ICAM‐1+ cells (Figure 1N,O) in both low‐ and high‐stimulation conditions in 3D co‐culture compared to 2D co‐culture. Thus, the presence of YK6 fibroblasts can boost B cell activation and proliferation in vitro.

### 2.2 Bleomycin‐Treated Fibroblasts Inhibit B Cell Proliferation, but Not Activation

We next established a system in which YK6 fibroblasts could be rendered senescent following a five‐day incubation with bleomycin, a DNA damaging agent (Jingyang Chen and Stubbe 2005). Bleomycin‐treated YK6 fibroblasts (Bleo‐YK6) upregulated senescence‐associated beta‐galactosidase (Figure 2A) and displayed features associated with senescence, as determined by flow cytometry (gated as in Figure S3): increased cell death (Figure 2B); increased forward (Figure 2C) and side (Figure 2D) scatter, suggesting cells are larger and more granular; and a reduction in proliferating cells, as measured by Ki67 staining (Figure 2E). At five days, Bleo‐YK6 cells had lower γ‐H2AX staining compared to controls (Figure 2F); however, they did show evidence of increased *IL6* and *CDKN1A* expression (Figure 2G), suggesting successful senescence induction. We confirmed this phenotype 24 h after bleomycin treatment, where bleomycin‐treated YK6 fibroblasts showed a similar, albeit more subtle, phenotype (Figure S2D–G). At this timepoint, however, YK6 fibroblasts had higher γ‐H2AX levels than controls (Figure S2G), suggesting recent DNA damage that leads to senescence.

**FIGURE 2:**
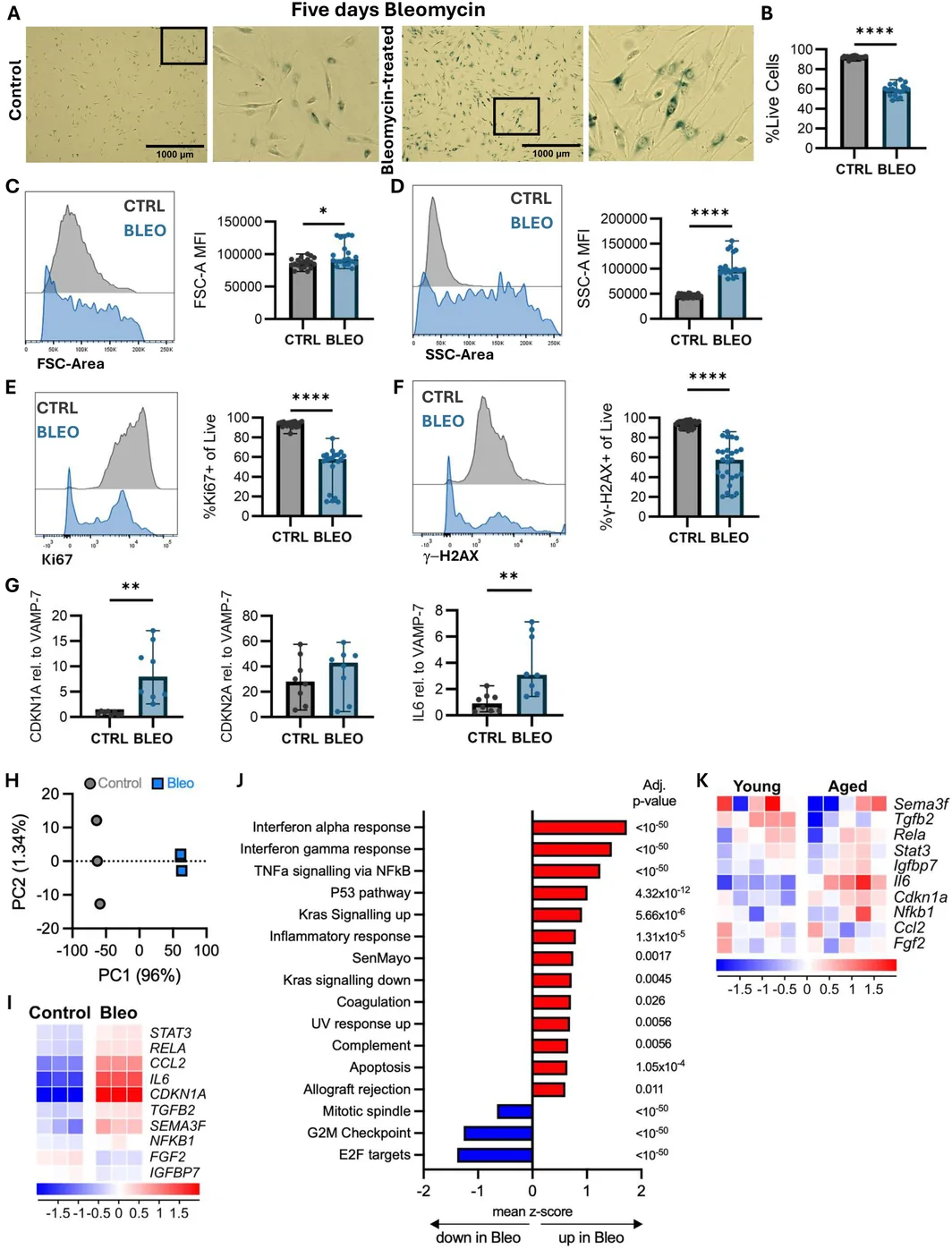
Bleomycin treatment induces cellular senescence in YK6 fibroblasts. (A–G) Analysis of YK6 fibroblasts after five days of bleomycin treatment. (A) SA‐β‐galactosidase staining on control (left) and bleomycin‐treated (right) YK6 fibroblasts. Scale bar 500 μm; boxed region is enlarged. (B–F) Flow cytometric analysis showing: the proportion of live cells (B); fibroblast size (FSC, C) and granularity (SSC, D); the proportion of proliferating cells (Ki67+, E); and the proportion of γ‐H2AX+ cells (F). Gating strategy shown in Figure S3A. (G) qRT‐PCR analysis, showing fold‐expression of *CDKN1A*, *CDKN2A* and *IL6* relative to *VAMP7*. Data are representative of five independent experiments with *n* = 5–6 senescent inductions per experiment. Statistical significance was determined with Mann–Whitney test, *p < 0.05, **p < 0.01, ****p < 0.0001. (H–J) Bulk RNA sequencing analysis of control‐ and Bleo‐YK6 fibroblasts showing Principal Component Analysis (H), expression (normalised log_2_(RPM)) of core senescence genes (I) and GSEA using the Hallmark gene sets (J). (K) Expression (normalised log_2_(RPM)) of core senescent genes in MRCs isolated from young (2‐month‐old) and aged (> 22‐month‐old) mouse lymph nodes. Mouse bulk RNAseq reflect five biological replicates, data reanalysed from (Denton et al. 2022).

To confirm induction of senescence, we performed bulk RNA sequencing of control and Bleo‐YK6 cells. Control and Bleo‐YK6 cells clustered separately by principal component analysis (Figure 2H), with upregulation of core senescence‐associated genes observed in Bleo‐YK6 cells (Figure 2I). Bleomycin treatment induced significant perturbation of the transcriptome, with > 18,000 genes differentially expressed when analysed by DESeq2. To understand the key changes, we performed gene set enrichment analysis (GSEA) using the Hallmark gene sets (Liberzon et al. 2015) alongside the SenMayo senescence gene set (Saul et al. 2022). Bleo‐YK6 fibroblasts had strongly downregulated cell cycle pathways (E2F targets, G2M checkpoint, Mitotic spindle; Figure 2J) compared to control YK6 fibroblasts, confirming cell cycle arrest previously determined by ki67 downregulation (Figure 2E). Upregulated pathways included SenMayo and several immune and inflammatory pathways (Figure 2J). To determine whether the senescent phenotype of Bleo‐YK6 cells phenocopied that of aged lymph node fibroblasts, we compared the bulk RNA sequencing DESeq2 results from control and Bleo‐YK6 with that of FDCs and MRCs purified from young and aged mice (Denton et al. 2022). There were few overlapping genes differentially expressed between aged MRCs and FDCs and there was little additional overlap with Bleo‐YK6 (Figure S2H), perhaps in part due to the high variability in the aged fibroblasts data given the low cell number inputs (Denton et al. 2022), or differences between mouse and human datasets. We did find that the SenMayo gene set was enriched by GSEA in aged mouse MRCs compared to those from younger adults (*z*‐score 0.059, *p* = 0.000239), and some key senescence‐associated genes such as *Il6*, *Cdkn1a* and *Igfbp7* were upregulated in aged MRCs (Figure 2K) akin to bleo‐YK6 (Figure 2I), suggesting that Bleo‐YK6 fibroblasts replicate some features of aged lymph node fibroblasts.

Having established a model for inducing senescence in YK6 cells, we next sought to determine how the B cell response was affected when co‐cultured in 2D with Bleo‐YK6 fibroblasts in no‐, low‐ and high‐stimulation conditions. We found that B cell proliferation, as measured by CTV dilution, was significantly reduced when B cells were co‐cultured with Bleo‐YK6 cells in both low‐ and high‐stimulation (Figure S4A–C). This was not, however, reflected in the expression of activation markers, where the proportion of B cells expressing CD69 (Figure S4D–F) and ICAM‐1 (Figure S4G–I) was not different between control and Bleo‐YK6 fibroblast co‐cultures. This data therefore suggests that Bleo‐YK6 fibroblasts specifically impede B cell proliferation. We noted a high degree of variability in the co‐cultures of B cells with control fibroblasts, which may be related to rapid growth of the fibroblasts; thus, we repeated these experiments using 3D co‐culture in Matrigel.

In 3D conditions, the B cell response when co‐cultured with Bleo‐YK6 cells was not significantly different to that observed with control YK6 cells in the absence of any stimulation, where B cell proliferation was not observed (Figure 3A). In contrast, B cell proliferation was essentially abrogated in 3D co‐culture with Bleo‐YK6 fibroblasts in both low‐ and high‐stimulation conditions (Figure 3B,C). B cell activation, determined by CD69 (Figure 3D) and ICAM‐1 (Figure 3G) expression, was not different between control and bleomycin‐treated YK6 co‐cultures in the absence of stimulation. In low‐ and high‐stimulation conditions, we observed a small but significant effect on the activation phenotype: in low‐stimulation conditions, there was no change in the proportion of B cells that were CD69+ when co‐cultured with Bleo‐YK6 fibroblasts, although there was a subtle increase in the level of its expression (Figure 3E). In contrast, the proportion of cells expressing ICAM‐1 was significantly lower in Bleo‐YK6 co‐cultures (Figure 3H). In high‐stimulation conditions there was a significant increase in the proportion of CD69+ B cells, as well as the level of CD69 expression (Figure 3F), and a subtle—but significant—decrease in the proportion of ICAM‐1+ (Figure 3I) B cells in Bleo‐YK6 co‐cultures compared to control YK6 co‐cultures. While this suggested an altered activation phenotype, our data in Figure 1 showed that CD69 was more highly expressed at earlier time points (Figure 1A–C), suggesting division‐linked expression. Indeed, when we assessed CD69 expression against CTV divisions, CD69 was more highly expressed at earlier divisions (Figure S5A), while ICAM‐1 was largely consistently expressed across divisions (Figure S5B). This suggests that B cells are equally activated when co‐cultured with Bleo‐ or control‐ YK6 fibroblasts but fail to progress through the cell cycle. To confirm this, we assessed the B cell phenotype 48 h after stimulation, prior to cell division (Figure S5C–E). We observed similar levels of activation in control and Bleo‐YK6 fibroblast co‐cultures, measured by both CD69 (Figure S5F–H) and ICAM‐1 (Figure S5I–K) expression. Therefore, these data suggest that Bleo‐YK6 fibroblasts specifically inhibit B cell proliferation.

**FIGURE 3:**
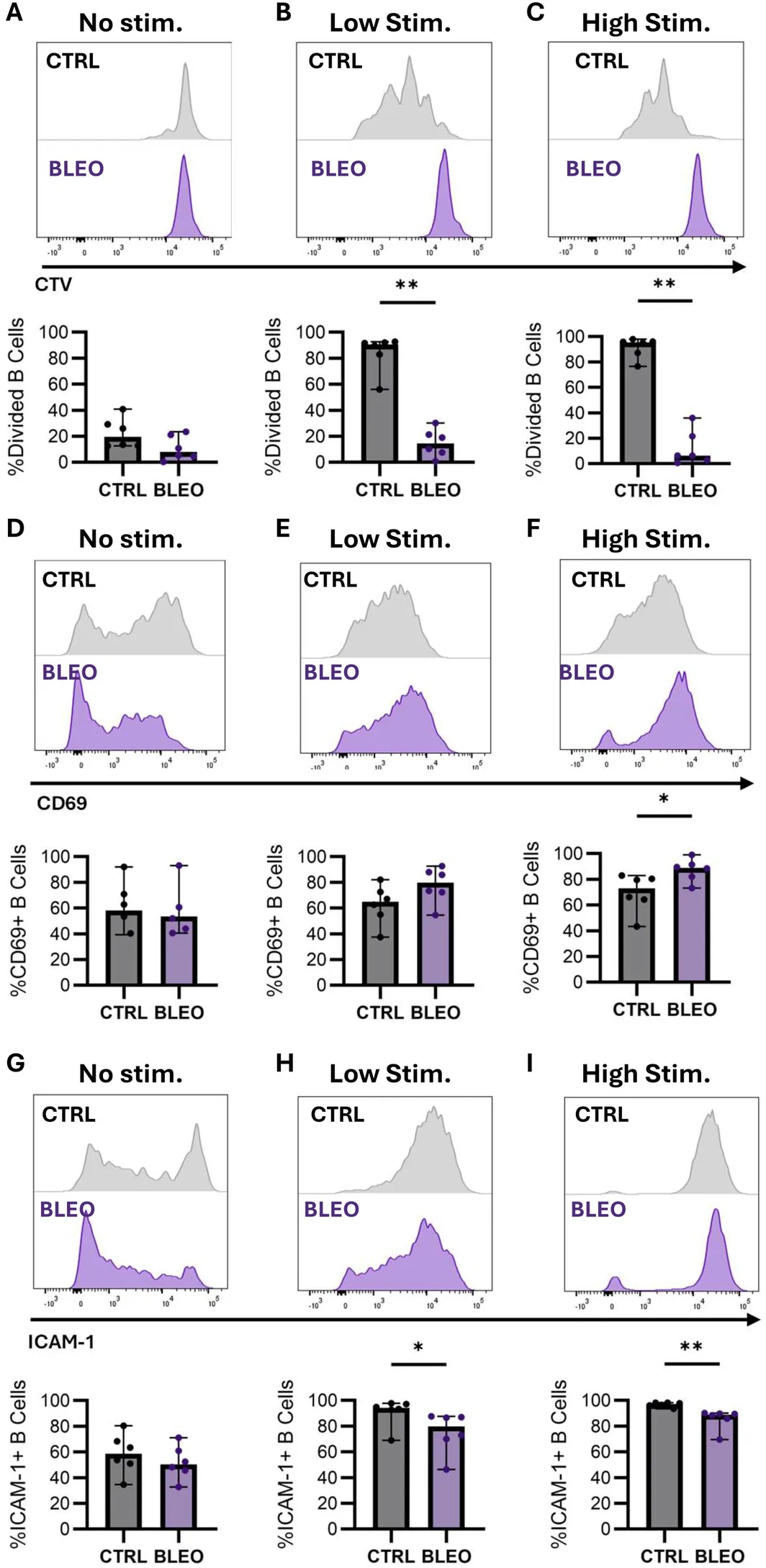
Co‐culture with bleomycin‐treated YK6 fibroblasts abrogates B cell proliferation. Naïve‐enriched PBMC‐derived B cells were co‐cultured with control or Bleo‐YK6 fibroblasts in 3D in Matrigel in no‐, low‐ or high‐ B cell stimulation conditions and analysed by flow cytometry four days later. (A–C) Representative flow cytometry histograms (top) and proportion of divided B cells (bottom) for no‐ (A), low‐ (B) and high‐ (C) stimulation conditions. (D–F) Representative flow cytometry histograms and proportion of CD69+ B cells for no‐ (D), low‐ (E) and high‐ (F) stimulation conditions. (G–I) Representative flow cytometry histograms and proportion of ICAM‐1+ B cells for no‐ (G), low‐ (H) and high‐ (I) stimulation conditions. Gating strategy shown in Figure S1A. Data are pooled from four independent experiments representing six PBMC donors. Statistical significance was determined with Mann–Whitney test, *p < 0.05, **p < 0.01.

### 2.3 Bleomycin‐Treated Fibroblasts Inhibit B Cell Proliferation Through Secreted and Cell Surface‐Expressed Proteins

Having established that Bleo‐YK6 fibroblasts inhibit B cell proliferation, we next sought to understand the mechanistic basis for this phenomenon. To this end we collected CM from control and five‐day bleomycin‐treated YK6 fibroblasts and stimulated B cells in the presence of control or Bleo‐YK6 CM. We first excluded the potential for a ‘senescence bystander effect’ (Waters et al. 2021) by incubating YK6 fibroblasts with CM from bleomycin‐treated YK6 cells for five days (Figure S6). We observed a subtle but statistically significant increase in YK6 fibroblast forward‐ and side‐scatter (Figure S6A,B), corresponding to the increased cell size and granularity, respectively. We also noted a small decrease in the expression of γ‐H2AX (Figure S6C) and Ki67 (Figure S6D), suggesting induction of a senescence‐like phenotype. Although statistically significant, these changes were substantially smaller than the phenotypic differences we observed in YK6 fibroblasts after five days of direct bleomycin treatment (Figure 2). Thus, we concluded that bleomycin was not leeching from cells, excluding senescence bystander effect.

We next assessed B cell activation and proliferation four days after no‐, low‐ and high‐stimulation in the presence of control or Bleo‐YK6 CM. In the absence of stimulation, no B cell proliferation or activation was observed (Figure 4A,D,G). In both low‐ and high‐stimulation conditions, Bleo‐YK6 CM led to a subtle, but significant, decrease in B cell proliferation (Figure 4B,C). B cells stimulated in the presence of Bleo‐YK6 CM were typically one division peak behind that of the control YK6 CM (Figure 4B,C), substantially different to the complete abrogation of B cell proliferation observed in Bleo‐YK6 co‐cultures (Figures S4B,C and 3B,C). The activation phenotype showed no significant difference in the proportion of CD69+ (Figure 4E,F) or ICAM‐1+ (Figure 4H,I) B cells in low‐ and high‐stimulation conditions (Figure 4E,F). Thus, we conclude that Bleo‐YK6 fibroblasts impair B cell proliferation largely through contact‐dependent mechanisms, with some contribution from secreted proteins.

**FIGURE 4:**
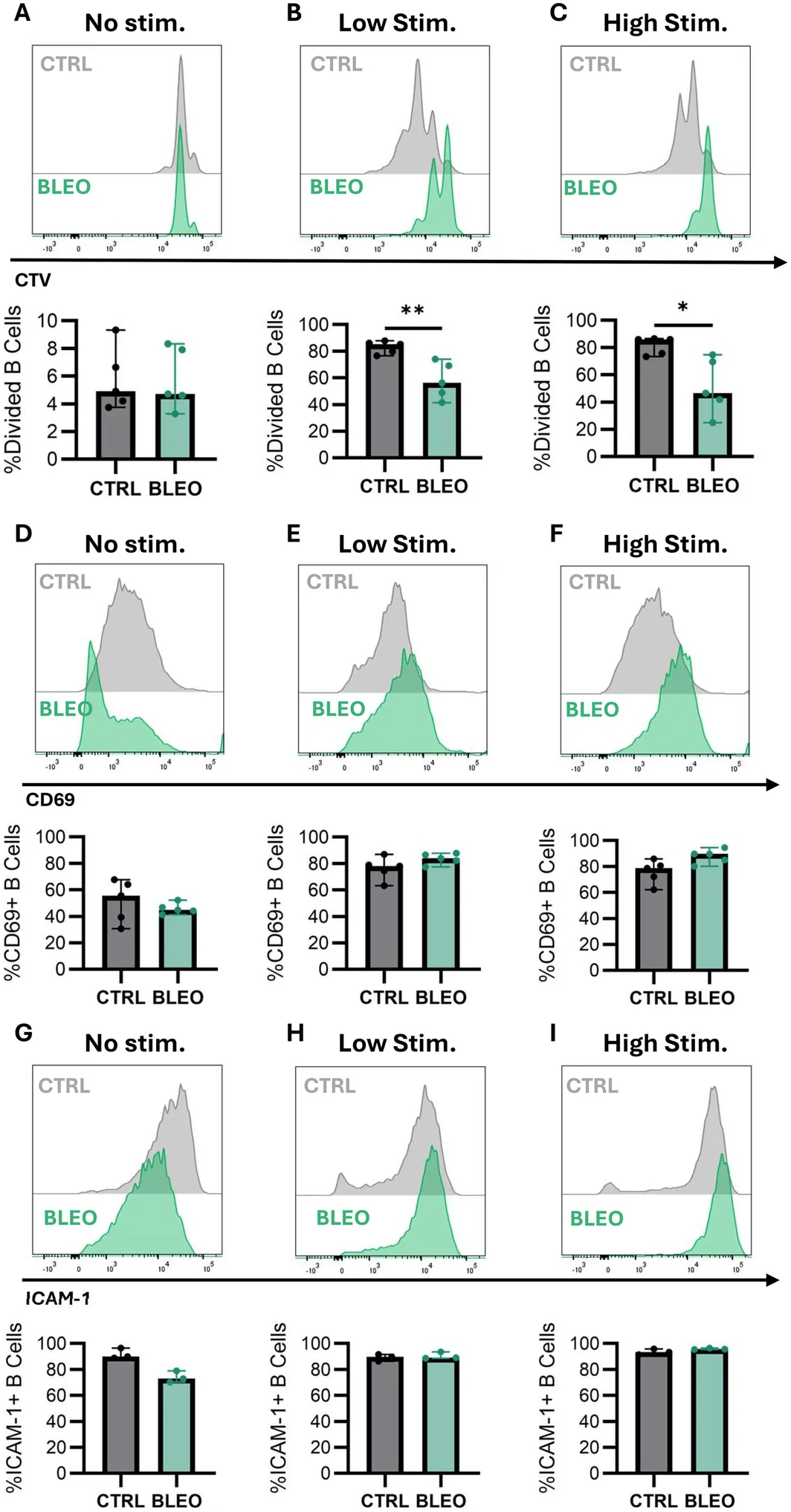
Bleomycin‐treated YK6 fibroblast‐derived conditioned media reduces B cell proliferation. Naïve‐enriched PBMC‐derived B cells were cultured in conditioned media from control or Bleo‐YK6 fibroblasts in no‐, low‐ or high‐ B cell stimulation conditions and analysed by flow cytometry four days later. (A–C) Representative flow cytometry histograms (top) and proportion of divided B cells (bottom) for no‐ (A), low‐ (B) and high‐ (C) stimulation conditions. (D–F) Representative flow cytometry histograms and proportion of CD69+ B cells for no‐ (D), low‐ (E) and high‐ (F) stimulation conditions. (G–I) Representative flow cytometry histograms and proportion of ICAM‐1+ B cells for no‐ (G), low‐ (H) and high‐ (I) stimulation conditions. Gating strategy shown in Figure S1A. Data are pooled from three independent experiments representing 5 PBMC donors. Statistical significance was determined with Mann–Whitney test, *p < 0.05, **p < 0.01.

### 2.4 Bleomycin‐Treated YK6 Fibroblasts Upregulate B Cell Inhibitory Ligands

We next sought to understand how bleomycin treatment altered YK6 fibroblasts, characterising the expression of B cell‐supportive transgenes introduced to YK6 fibroblasts (CD40L and BAFF) five days after bleomycin treatment. The expression of introduced transgenes was also reduced by bleomycin treatment (Figure 5A,B), with fewer cells expressing CD40L and BAFF (marked by mouse CD8α expression). Bleo‐YK6 fibroblasts also expressed lower levels of CD40L and BAFF, as indicated by the geometric mean fluorescence intensity (gMFI) of antibody staining (Figure 5A,B), and the secretion of BAFF into the culture media was also reduced (Figure 5C). A subtle reduction in CD40L and BAFF levels was also observed when YK6 fibroblasts were cultured in senescent CM (Figure S6F,G). Because bleomycin treatment alters YK6 fibroblast size and growth characteristics, which may affect the flow cytometric measurements, we also assessed surface marker expression 24 h after bleomycin treatment. CD40L gMFI was not altered at 24 h (Figure S7A), while there was a small increase in BAFF, as measured by CD8α expression (Figure 5B). To better understand how bleomycin treatment altered YK6 cells, we analysed the expression of cell surface molecules that have previously been associated with fibroblast‐B cell interactions in our Bleo‐YK6 bulk RNA sequencing. We found increased expression of *ICAM1*, *CD274* (PD‐L1), *CD74*, *TNFRSF14* (HVEM), and *PDCD1LG2* (PD‐L2) transcripts in Bleo‐YK6 fibroblasts, while *NT5E* (CD73) was slightly reduced (Figure 5E). Increased ICAM‐1 (by gMFI) was also observed by flow cytometry five days after bleomycin treatment (Figure 5E)—although slightly fewer cells were ICAM‐1+ overall. ICAM‐1 gMFI was also increased after just 24 h of bleomycin treatment (Figure S7C), suggesting upregulation of ICAM‐1 may be a direct response to the DNA damage. Bleomycin treatment also led to reduced cell surface expression of CD73 (Figure 5F). Together these data suggest that bleomycin may have induced or increased expression of negative regulators of B cell activation and/or proliferation.

**FIGURE 5:**
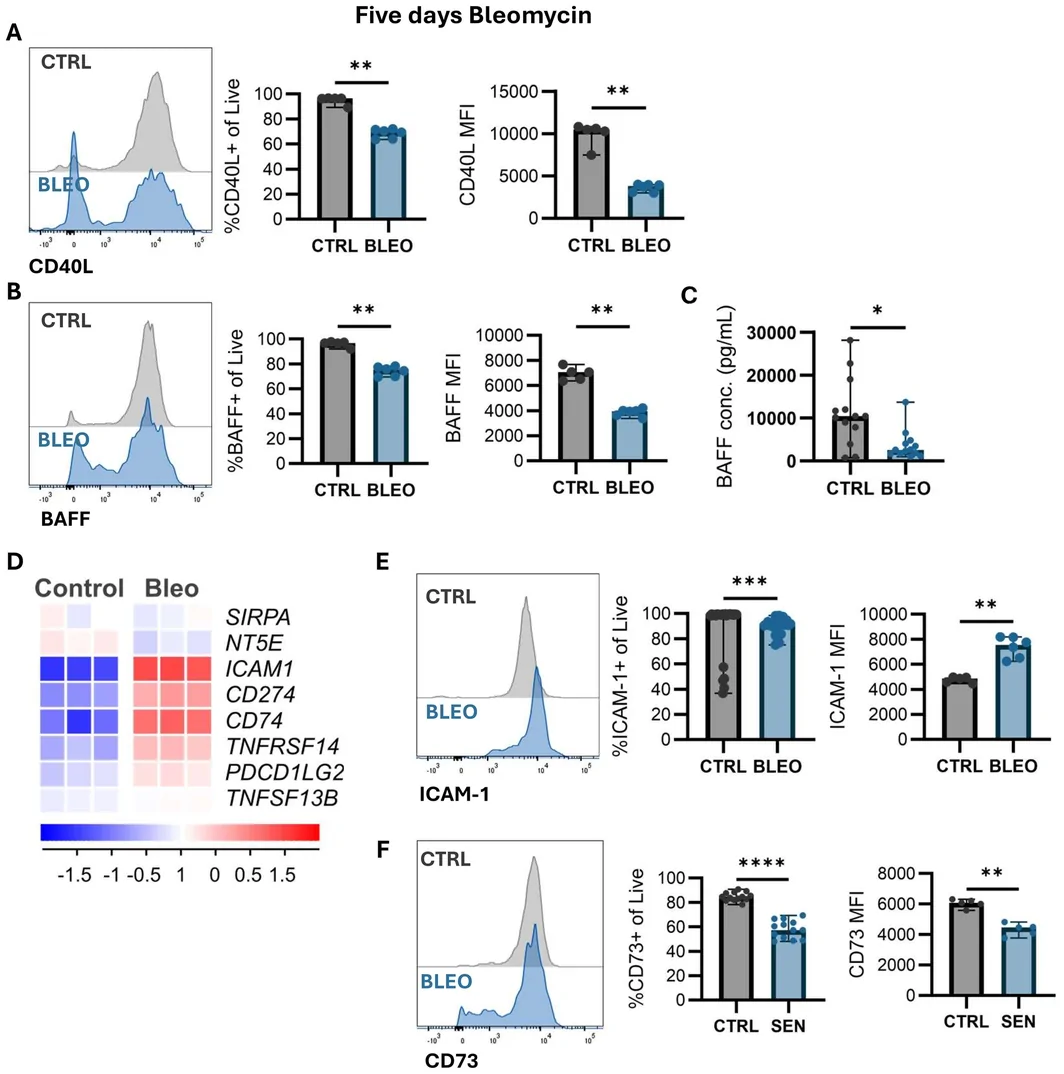
Bleomycin treatment alters expression of B cell‐interacting ligands by YK6 fibroblasts. YK6 fibroblasts were treated with bleomycin for five days and their phenotype determined by flow cytometry and RNA sequencing. (A, B) The proportion and gMFI of CD40L (A) and BAFF (B) were determined by flow cytometry. (C) The concentration of BAFF was determined in control and Bleo‐YK6 fibroblast culture media via ELISA. (D) Heatmap showing expression of B cell‐interacting genes by control and Bleo‐YK6 fibroblasts. Data are shown in log_2_(RPM), normalised within each gene. (E, F) The proportion and gMFI of ICAM‐1 (E) and CD73 (F) were determined by flow cytometry. Gating strategy shown in Figure S3. Data in (A–C, E, F) are representative of five independent experiments with *n* = 5–10. Statistical significance was determined with Mann–Whitney test, *p < 0.05, **p < 0.01, ***p < 0.001, ****p < 0.0001.

### 2.5 Co‐Culture With Bleo‐YK6 Cells Restrains Metabolic Rewiring of B Cells

While cell surface markers indicated that B cells were activated in the presence of Bleo‐YK6 fibroblasts (Figure 3), we next sought to determine whether B cells had undergone activation‐driven switch to glycolysis (Mosaffa Jahromi et al. 2026) in our 3D co‐culture system. B cells co‐cultured with control YK6 fibroblasts readily increased expression of GLUT1 (Figure 6A) and CD98 (Figure 6B), compared to unstimulated, non‐cultured naïve‐enriched B cells. However, B cells co‐cultured with Bleo‐YK6 fibroblasts, when compared to B cells co‐cultured with control YK6 fibroblasts, were impaired in their ability to upregulate GLUT1 (Figure 6C), although a similar proportion of B cells upregulated CD98 in control‐ and Bleo‐YK6 co‐cultures (Figure 6D). We next determined whether co‐culture with Bleo‐YK6 cells affected protein synthesis by measuring puromycin incorporation. To control for differences in B cell activation state, we gated on undivided, activated (MHCII^hi^ CD69+) B cells in both control and Bleo‐YK6 co‐cultures (Figure S1B,C). While B cells cultured with control YK6 cells incorporated puromycin at high levels, this was significantly lower when B cells were co‐cultured with Bleo‐YK6 cells (Figure 6E).

**FIGURE 6:**
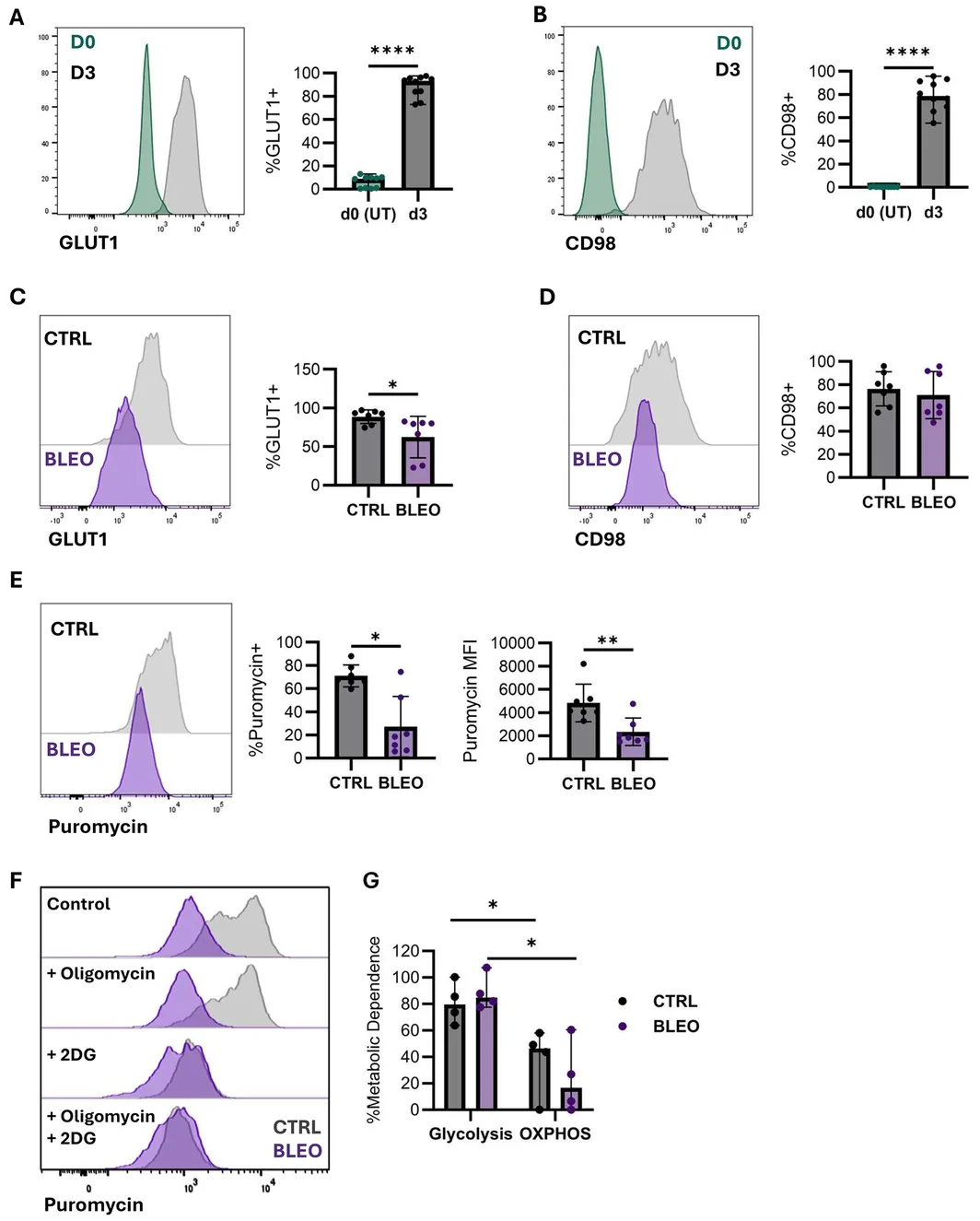
Co‐culture with Bleo‐YK6 cells arrests the metabolic rewiring of B cells following activation. (A, B) Representative flow cytometry histograms and proportion of B cells expressing GLUT1 (A) and CD98 (B), comparing unstimulated, non‐cultured cells (d0) and B cells co‐cultured with control YK6 cells (d3). (C, D) Naïve‐enriched PBMC‐derived B cells were co‐cultured with control or Bleo‐YK6 fibroblasts in 3D in high stimulation conditions for three days and analysed by flow cytometry, gated as in Figure S1B,C. Representative flow cytometry histograms (left) and proportion (right) of B cells expressing GLUT1 (C) and CD98 (D), comparing between B cells co‐cultured with control‐ or Bleo‐YK6. (E–G) Naïve‐enriched PBMC‐derived B cells were co‐cultured with control‐ or Bleo‐YK6 fibroblasts in 3D in high stimulation conditions for three days and puromycin incorporation determined in the absence (E), or presence (F, G) of mitochondrial inhibitors (SCENITH assay). Representative flow cytometry histograms (left), proportion of puromycin+ B cells (middle) and puromycin gMFI, gated on all B cells (right) (E). Gated as in Figure S1A. Representative flow cytometry histograms of puromycin gMFI from MHC II^hi^CD69+ undivided B cells (gating in Figure S1B,C) cultured with control‐ (grey) or bleo‐ (purple) YK6 cells (F). B cell metabolic dependence on glycolysis or OXPHOS, calculated as % dependence (G). Data are pooled from 3 to 4 independent experiments representing 4–7 PBMC donors. Statistical significance was determined with Mann–Whitney tests, *p < 0.05, **p < 0.01, ****p < 0.0001.

To determine whether B cells co‐cultured with control or Bleo‐YK6 cells were using glycolysis or oxidative phosphorylation (or both), we performed the SCENITH assay (Argüello et al. 2020). We analysed undivided, activated (MHCII^hi^ CD69+) B cells, as before, and determined dependence on glycolysis and oxidative phosphorylation (OXPHOS) through inhibition with 2‐deoxy‐D‐glucose (2‐DG) and/or oligomycin A, respectively. Stimulated B cells were almost totally reliant on glycolysis and were not using OXPHOS regardless of the presence of control or Bleo‐YK6 cells in the co‐culture (Figure 6F,G). It is important to note, however, that the level of puromycin incorporation in Bleo‐YK6 co‐cultures was significantly lower than control co‐cultures across all metabolic conditions (Figure 6F), suggesting that the ‘activated’ B cells in co‐culture with Bleo‐YK6 fibroblasts are preferentially using glycolysis, albeit with poor synthesis of proteins (Figure 6E–G). Taken together, our data suggest while the metabolic preferences of B cells were largely unchanged, exposure to bleomycin‐treated YK6 fibroblasts induced a failed activation state characterised by poor upregulation of proteins that support glycolysis and significant impairment of protein synthesis.

## 3 Discussion

In this study we set out to understand how fibroblast senescence may impact the ability of fibroblasts to support the humoral response. Using YK6 fibroblasts, a human tonsil‐derived B zone reticular‐like cell line engineered to express membrane‐bound CD40L and secrete BAFF, we established that lymph node‐derived fibroblasts support B cell activation in vitro; however, upon treatment with bleomycin, YK6‐supported B cell proliferation was abrogated, characterised by partial engagement of glycolysis and an inability to synthesise proteins despite displaying an activated phenotype. The impairment of the B cell response was not due to secreted factors, including a bystander senescence effect, suggesting that cell–cell interactions directly impair B cell proliferation in this setting. Our data suggest that upregulation of negative regulators of B cell activation in response to bleomycin treatment may play a role in the impaired B cell response. Our results suggest a hitherto unexpected impact of fibroblast senescence on the ability of B cells to participate in immune reactions.

Our results demonstrate that fibroblast senescence affects B cell proliferation and capacity to synthesise new proteins, whilst showing features of having been activated. The inability to proliferate despite adequate activation is reminiscent of chronic lymphocytic leukaemia, where B cells are unable to respond even to mitogenic stimulation with phorbol myristate acetate (PMA) (Engel et al. 1989), suggestive of failed calcium signalling. Several receptors have been described in limiting B cell proliferation, including PD‐1, Fas, BTLA, and FcγRIIB (Strasser et al. 2009; Thibult et al. 2013; Watanabe et al. 2003; Xu et al. 2014). While YK6 fibroblasts did not express canonical FDC markers such as CR1/2 and FcγRIIB, bleomycin treatment did lead to the upregulation of ligands for some B cell receptors, including *CD274* (PD‐L1), *TNFRSF14* (HVEM), and *PDCD1LG2* (PD‐L2). Additionally, ICAM‐1 was increased at the protein level on bleomycin‐treated YK6 fibroblasts, suggesting that senescent fibroblasts may directly inhibit B cell activation via cell–cell contact. Increased ICAM‐1 on Bleo‐YK6 fibroblasts may contribute to the B cell phenotype observed, as others have noted that high expression of ICAM‐1 on T cells impairs B cell activation and immunoglobulin class switching (Liu et al. 2023). We noticed in Bleo‐YK6 co‐cultures that B cells were not in the classic ‘proliferative clusters’ typically formed by lymphocytes and were largely sitting along fibroblast processes. Whether the increased ICAM‐1 expression by fibroblasts may ‘tether’ B cells to the fibroblasts, which could increase their exposure to, or signalling via, negative regulators such as PD‐L2 and/or CD73, remains to be determined.

An outstanding question is how relevant these findings are to human ageing. In the absence of an aged human lymph node fibroblast RNA sequencing dataset, we attempted to find similarities between aged mouse lymph node fibroblasts and bleomycin‐treated YK6 fibroblasts. Very few similarities were observed, outside of both aged mouse MRCs and Bleo‐YK6 cells being enriched for the SenMayo gene sets; thus, our results should be interpreted with some caution, especially given that the YK6 cells more closely resemble B zone reticular cells and lack key FDCs markers such as CR1 and FcγRIIB. Age‐associated reductions in FDC‐expressed antigen‐presenting machinery, such as Cr1/2 and FcγRIIB, impair the ability of FDCs to support the GC response (Aydar et al. 2004; Szakal et al. 1988, 1990). Of note, loss of FcγRIIB on aged FDCs may be linked mechanistically to impaired B cell proliferation in ageing, as FcγRIIB‐deficient FDCs inhibit BCR signalling via disruption of SHIP phosphorylation (Aydar et al. 2004), thus reducing B cell proliferation. Absence of FcγRIIB on FDCs in younger adult mice also increases the breadth of selection, increasing GC B cell diversity (van der Poel et al. 2019), leading to epitope spreading and autoimmunity, akin to the association between FcγRIIB polymorphisms and autoimmunity in humans (Chu et al. 2004; Kyogoku et al. 2002). Development of autoimmunity is also associated with ageing (Rowley et al. 1968), suggesting that disruption of FcγRIIB expression on FDCs may underpin some of the age‐associated changes in the lymph node that impact on humoral immunity.

Numerous studies have demonstrated that age impacts the humoral response (Chen et al. 2022; Frasca et al. 2011), and recent studies emphasise a significant role for immune‐extrinsic ageing. Here we describe an in vitro system in which senescent FDCs directly impaired the proliferative capacity of human B cells, highlighting a potential mechanism by which the immune system may be rendered dysfunctional in ageing. The exact mechanistic underpinning of these results remains elusive; further analyses of the aged immune microenvironment will likely shed light on these phenomena, particularly in the emerging era of single cell data.

## 4 Methods

### 4.1 YK6 Fibroblast Culture, Senescence Induction and Conditioned Media

YK6 fibroblasts (Caeser et al. 2021) were cultured in glutaMAX DMEM (Thermofisher, #31966‐021) supplemented with 10% FBS (PAN Biotech, #P30‐193031) and 1% penicillin/streptomycin (Sigma‐Aldrich, #P0781‐100ML). YK6 fibroblasts were seeded at 2 × 10^5^ cells/well on 6‐well plates, 10^5^ cells/well on 12‐well plates, or 5 × 10^6^ cells/T175 flask. Senescence was induced with 50 μg/mL bleomycin (Cayman Chemical, #CAY13877) for 5 days, after which cells were washed three times with media and reseeded 0.3 × 10^5^ cells/well for co‐culture with B cells. To generate CM, YK6 fibroblasts were subjected to bleomycin treatment for five days, washed and cultured in fibroblast media for an additional four days to reflect the B cell co‐culture period, after which CM was collected, centrifuged at 600 rcf for 6 min and stored at −20°C. For control CM, media was collected from standard YK6 culture media prior to splitting and feeding and stored at −20°C.

### 4.2 Senescence‐Associated Beta‐Galactosidase Staining

YK6 fibroblasts were seeded on 6‐well plates and treated with bleomycin for five days and stained with the senescence‐associated beta‐galactosidase staining kit (Cell Signalling Technology, cat. #9860), following the manufacturer's instructions. Images were obtained with a Leica DM4 brightfield microscope at 4× magnification.

### 4.3 PBMC Processing

Leucocyte cones were obtained from the NHSBT‐NCI service; each cone was sourced from a single human donor of unknown age and sex. Peripheral Blood Mononuclear Cells (PBMCs) were purified by density separation by centrifugation over Lymphoprep (STEMCELL Technologies, #07851). Briefly, each cone was diluted with PBS/2% FBS at room temperature to a total of 80 mL. 20 mL of blood was layered on 15 mL Lymphoprep and centrifuged at 800× *g* for 20 min at room temperature without brake. The PBMC layer was collected, washed twice with PBS/2% FBS, and stored in liquid nitrogen.

### 4.4 Naïve B Cell Isolation Stimulation, Co‐Culture and CM Experiments

PBMCs were thawed and naïve‐enriched B cells were isolated using the Naïve B Cell Isolation Kit II human (Miltenyi Biotec, #130‐091‐150) with LS columns (Miltenyi Biotec, #130‐042‐401), according to manufacturer's instructions. Purified naïve‐enriched B cells were adjusted to 10^6^ cells/mL and labelled with 0.25 μM CTV (Invitrogen, #C34557) at 37°C for 20 min, according to manufacturer's instructions and cultured in complete (c)RMPI media containing 10% FBS, 2 mM L‐glutamine (Sigma‐Aldrich, #G7513), 1× penicillin–streptomycin (Sigma‐Aldrich, #P0781), and 50 μM 2‐Mercaptoethanol (Thermofisher, #31350‐010). 3 × 10^5^ B cells were cultured in 500 μL cRPMI in 48 well plates stimulated with 1.5 μg/mL soluble CD40 ligand (Peprotech, cat. #310‐02), 15 μg/mL anti‐IgM (Jackson ImmunoResearch, #109‐005‐129), and 5 μg/mL CpG (InvivoGen, #tlr‐2395). High stimulation conditions were additionally supplemented with 100 ng/mL IL‐2 (Peprotech, #200‐02), 10 ng/mL IL‐4 (Peprotech, #200‐04), and 10 ng/mL IL‐6 (Peprotech, #200‐06). Unstimulated controls were included in all conditions. Cells were cultured at 37°C 5% CO_2_ for up to seven days. For the CM experiments, stimulation mixes were instead made in CM from control or bleomycin‐treated YK6 cells, with reagent concentrations calculated as before for 500 μL mix/well. 490 μL of the appropriate stimulation mix were added to each well, followed by 3 × 10^5^ B cells/well in 10 μL of fresh media, to ensure final concentrations of all reagents remain unchanged.

### 4.5 2D and 3D Co‐Culture

For 2D co‐culture, YK6 fibroblasts were treated with bleomycin five days before co‐culture to generate senescent YK6 fibroblasts, while control YK6 fibroblasts were seeded one day prior to co‐culture in 48‐well plates. Media was removed from control and Bleo‐YK6 monolayers and replaced with 500 μL/well of the appropriate stimulated B cell mix, prepared as described above (3 × 10^5^ cells/well). For 3D co‐culture, 48‐well plates were first pre‐chilled overnight at −20°C, then coated with 150 μL of thawed Matrigel Basement Membrane Matrix (Corning, cat. #354234). Coated plates were incubated at 37°C for 30 min to allow the gel to solidify, then seeded using the same process as above. B cells were stimulated for two or four days and proliferation and activation assessed by flow cytometry.

### 4.6 Flow Cytometry

Cultured B cells and/or fibroblasts were harvested and placed in 5 mL polystyrene round‐bottom tubes, washed with 1 mL PBS/2% FBS/2 mM EDTA and pelleted at 400× *g* for 5 min at room temperature. B cells ± YK6 fibroblasts were blocked with 0.5 μL of human TruStain FcX block (Biolegend, #422302) for 5 min, then stained with 50 μL of appropriate antibody mix (Table 1) for 30–45 min at 4°C. Cells were washed, fixed with 100 μL of 2% PFA (Sigma‐Aldrich, #158127) then washed, and data collected on a BD Fortessa X‐20. YK6 fibroblasts were stained with 100 μL of appropriate antibody mix (Table 2) for 30–45 min at 4°C. Cells were fixed with 100 μL of Foxp3 Transcription Factor Staining kit (Thermofisher, #00‐5523‐00) for 30 min at 4°C, washed with 1× Perm/Wash buffer and stained intracellularly with antibodies (Table 2) overnight at 4°C. Cells were finally washed and data collected on a BD Fortessa X‐20.

**TABLE 1:** Flow cytometry antibodies for B cell phenotyping.

Marker	Fluorochrome	Clone	Manufacturer	Cat. No.	Dilution
Viability stain	efluor 780	—	Invitrogen	65‐0865‐14	1:1000
ICAM‐1	APC	HA58	Biolegend	353112	1:200
CD19	PE	HIB19	Biolegend	302208	1:100
CD19	BV 605	HIB19	Biolegend		1:100
CD20	FITC	2H7	Biolegend	302304	1:200
CD69	BV 605	FN50	Biolegend	310938	1:100
CD69	BV 785	FN50	Biolegend		1:100
CD98	PE	UM7F8	BD Biosciences	556077	1:100
GLUT1	AF 488	EPR3915	BD Biosciences	569321	1:100
Puromycin	AF 647	12D10	Sigma‐Aldrich	MABE343‐AF647	1:100
HLA‐DR (MHC II)	PE‐Cy7	Tü39	Biolegend	361708	1:1000

**TABLE 2:** Flow cytometry antibodies for YK6 phenotyping.

Antibody marker	Fluorochrome	Clone	Manufacturer	Cat. No.	Dilution
Viability stain	efluor 780	—	Invitrogen	65‐0865‐14	1:1000
CD44	AF 700	IM7	Biolegend	103026	1:1000
ICAM‐1 (CD54)	BV 421	HA58	BD Biosciences	564077	1:1000
Podoplanin	PE‐Cy7	NC‐08	Biolegend	337014	1:1000
CD40L (CD154)	APC	24–31	Biolegend	310809	1:400
mouseCD8a (for BAFF)	PerCP‐Cy5.5	53–6.7	Biolegend	100734	1:400
gH2A.X Phospho (Ser139)	PE	2F3	Biolegend	613412	1:2000
Ki67	FITC	SolA15	Invitrogen	11‐5698‐82	1:2000
Ki67	AF 700	SolA15	Invitrogen		1:1000
CD73	BV 510	AD2	Biolegend	344044	1:300
PD‐L2 (CD273)	AF 488	24F.10C12	BD Biosciences	569409	1:100
CD32b/c	BV 605	S18005H	Biolegend	398338	1:100
CD23	PE‐Cy7	EBVCS‐5	Biolegend	338516	1:100
Cr1 (CD35)	PE	E11	BD Biosciences	559872	1:100
L1CAM (CD171)	AF 647	L1‐OV198.5	Biolegend	371608	1:100

### 4.7 Puromycin Incorporation and SCENITH Assay

PBMC‐derived B cells were co‐cultured with control or Bleo‐YK6 cells as described above in hi‐stimulation conditions for three days. 10 μg/mL puromycin (Sigma‐Aldrich, #P8833‐10MG) was added to all culture wells and incubated at 37°C, 5% CO_2_ for 15 min. All samples were subsequently harvested and prepared for flow cytometry staining as described above, with the addition of an anti‐Puromycin antibody (Sigma‐Aldrich, #MABE343‐AF647). Metabolic activity was calculated by measuring the gMFI of anti‐puromycin staining. The SCENITH assay was performed essentially as described (Argüello et al. 2020). Briefly, the metabolic inhibitors 2‐Deoxy‐Glucose (2DG; Sigma‐Aldrich, #D6134‐25G) or Oligomycin A (Sigma‐Aldrich, #75351‐5MG), or a mixture of both, were added to the co‐cultured B cells at final concentrations of 100 mM and 1 μM respectively for 15 min prior to the addition of puromycin. For analysis of puromycin gMFI, technical duplicates for individual PBMC donors were averaged within each experiment. Metabolic dependence on OXPHOS or Glycolysis was calculated as described in (Argüello et al. 2020).

### 4.8 BAFF Elisa

Supernatants collected from control and bleomycin‐treated YK6 fibroblasts were diluted 1:100 and BAFF concentration determined using the human BAFF ELISA MAX Deluxe set (Biolegend, #449704) per manufacturer's instructions. Developed ELISA plates were measured at 450 nm, 570 nm using a Multiskan FC Microplate Photometer (ThermoFisher).

### 4.9 Quantitative RT‐ PCR

RNA from 0.5‐1 × 10^7^ cells was purified using the RNeasy Mini Kit (Qiagen, cat. no. 74104) following the manufacturer's instructions. RNA concentration was quantified using a NanoDrop One (Thermo Scientific) and 1 μg RNA converted into cDNA using the iScript cDNA Synthesis Kit (Bio Rad, #1708891) and stored at −20°C until use. Taqman primer‐probes were used for qPCR: *CDKN2A* (Hs00923894_m1), *CDKN1A* (Hs00355782_m1) and *IL6* (Hs00174131_m1) were assayed alongside *VAMP7* (Hs00194568_m1) and *L3MBT2L* (Hs01002034_m1) as housekeeper genes. qPCRs were conducted in 10 μL reactions using 50 μg of cDNA template in TaqMan Fast Advance Master Mix (Applied Biosystems, #4444554) using the ViiA 7 Real‐Time PCR System (Applied Biosciences). Ct values were converted to an arbitrary copy number using the equation: copy number = (10^5^)/(2^Ct‐17^). Gene expression was determined relative to housekeeper genes.

### 4.10 Bulk RNA Sequencing and Analysis

Total RNA was purified from control and Bleo‐YK6 cells as described above and RNA quality (RIN > 9) was confirmed with a bioanalyzer (Agilent RNA 6000 Nano Kit, #5067‐1511). RNA library preparation and sequencing were performed by GENEWIZ. Briefly, RNA libraries were prepared with the NEBNext Ultra II RNA Library prep kit (NEB #E7775), mRNAs were enriched with poly(A) selection, and 150 bp paired‐end sequencing conducted using an Illumina NovaSeq X. Bam files, aligned to GRCh38 (STAR aligner v2.5.2b) by GENEWIZ, were analysed in Seqmonk (version 1.49.1; www.bioinformatics.babraham.ac.uk/projects/seqmonk/). Reads were quantitated and library size was standardised to reads per million after non‐annotated genes (‘novel transcript’) were removed. Expressed genes were defined as having log_2_(RPM) > −4 in all replicates. Differentially expressed genes (*p* < 0.05, Bejamimi & Hochberg correction) between control and Bleo‐YK6 were determined using Seqmonk's inbuilt DESeq2 analysis. GSEA was conducted using Hallmark (h.all.v2026.1) and Senmayo (SAUL_SENMAYO.v2026.1) human gene sets. Bulk RNA sequencing of mouse young and aged FDCs and MRCs, analysed as in Denton et al. (2022), were subjected to additional analyses. Genes up‐ or downregulated in aged FDCs and MRCs, compared to younger adult controls, were extracted from DESeq2 analysis conducted as described in Denton et al. (2022). Overlaps between genes up‐ or downregulated in MRCs or FDCs and genes up‐ or down‐regulated in Bleo‐YK6 cells were determined and data displayed using DeepVenn (deepvenn.com; Hulsen 2022). GSEA on aged mouse FDCs and MRCs was conducted using the Senmayo (SAUL_SENMAYO.v2026.1) mouse gene set. All transcriptomic data are available in Data S1. Gene expression data are available through the Gene Expression Omnibus: Mouse FDC and MRC GSE150054; control and Bleo‐YK6 GSE346528.

### 4.11 Data and Statistical Analysis

Data were graphed and statistical analysis was performed on GraphPad Prism v10. Statistical significance was conducted using non‐parametric tests: Mann–Whitney tests to compare two groups and Kruskal‐Wallis tests with Dunn's multiple comparisons to compare three or more groups, *p < 0.05, **p < 0.01, ***p < 0.001, ****p < 0.0001.

## Author Contributions

**Kassandra Vezyrgianni:** formal analysis, investigation, methodology, supervision, writing – original draft, writing – review and editing. **Elizabeth MacLeod:** investigation. **Abigail Nutley:** investigation, methodology. **Yuchen Zhu:** methodology. **Alice E. Denton:** conceptualisation, funding acquisition, supervision, writing – original draft, writing – review and editing.

## Funding

Work was supported by funding from the Dunhill Medical Trust (AIS2110\20) and a UKRI MRC Career Development Award (MR/V009591/1) to AED.

## Conflicts of Interest

The authors declare no conflicts of interest.

## Supporting information


**Data S1:** Supporting Information.

**Figure S1–S7:** acel70766‐sup‐0003‐FigureS1‐S7.pdf

## Data Availability

Gene expression data used in this publication are available through the Gene Expression Omnibus (https://www.ncbi.nlm.nih.gov/geo/): mouse FDC and MRC GSE150054; control and Bleo‐YK6 fibroblasts GSE346528.
