# Supplementary material for: Senescent Fibroblasts Impair B Cell Proliferation via Cell–Cell Contact
Source: Aging Cell. 2026 Oct 16;25(10):e70766. doi: 10.1111/acel.70766 (PMC13673512; doi:10.1111/acel.70766)
Supplement: Supplementary file 1 [file ACEL-25-e70766-s001.pdf]

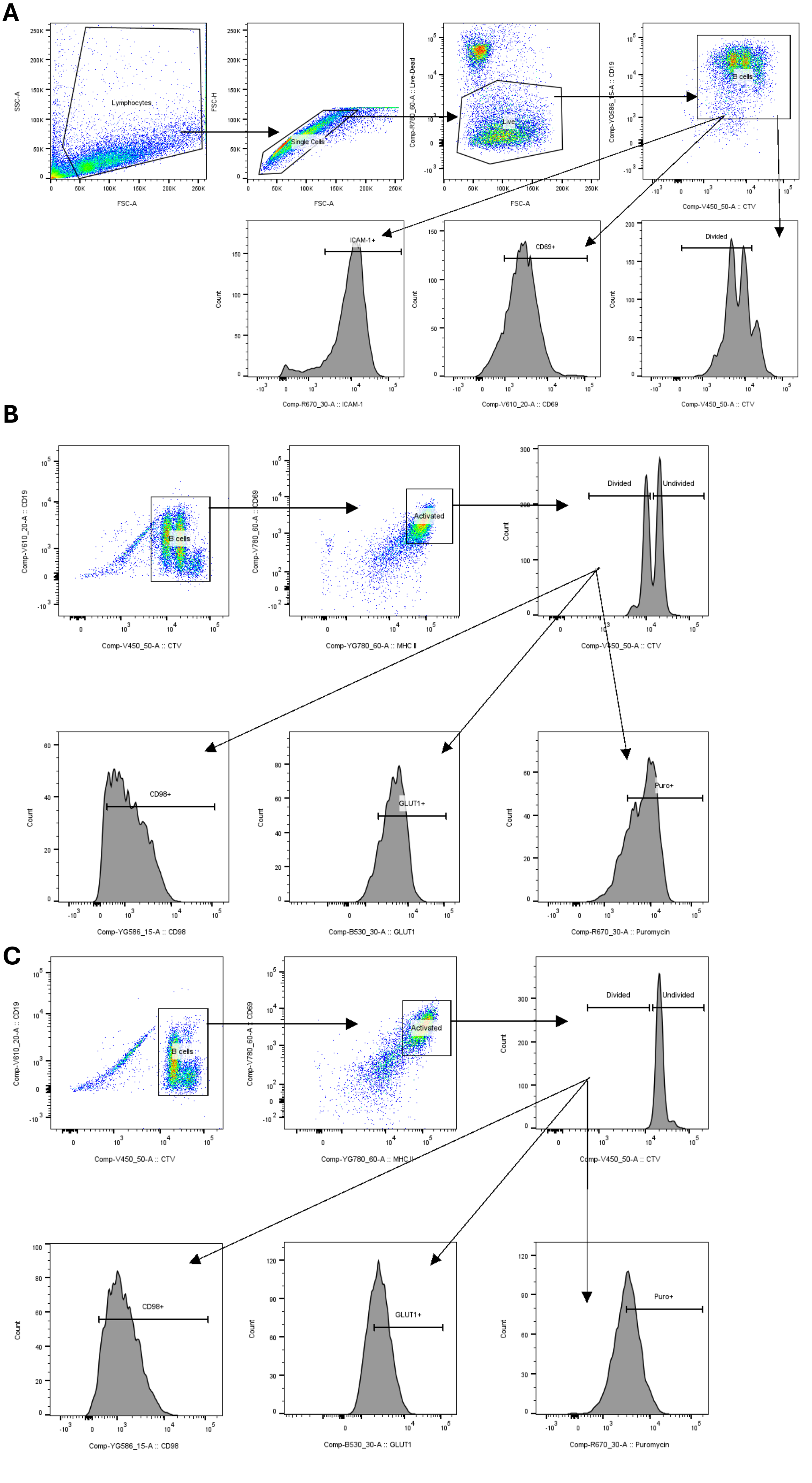


**Figure S1: Flow cytometry gating of stimulated B cells.** (**A**) Gating for B cell activation. B cells were gated as lymphocytes, single cells, live cells, then CTV+CD19+. Divided B cells were determined by CTV dilution, compared to CTV labelled, enriched cells (no stimulation or culture). ICAM-1- and CD69-expressing populations were determined by unstained controls. (**B-C**) Gating for metabolic evaluation of B cells. B cells were gated as lymphocytes, single cells, live cells, then CTV+CD19+. Activated B cells were defined as MHC II$^{hi}$, CD69+ cells. Undivided, activated were determined by CTV labelling. Expression of CD98 and GLUT1 and incorporation of puromycin were determined using unstained controls. Gating B cells co-cultured with control (**B**) and Bleo-YK6 (**C**) cells are shown.

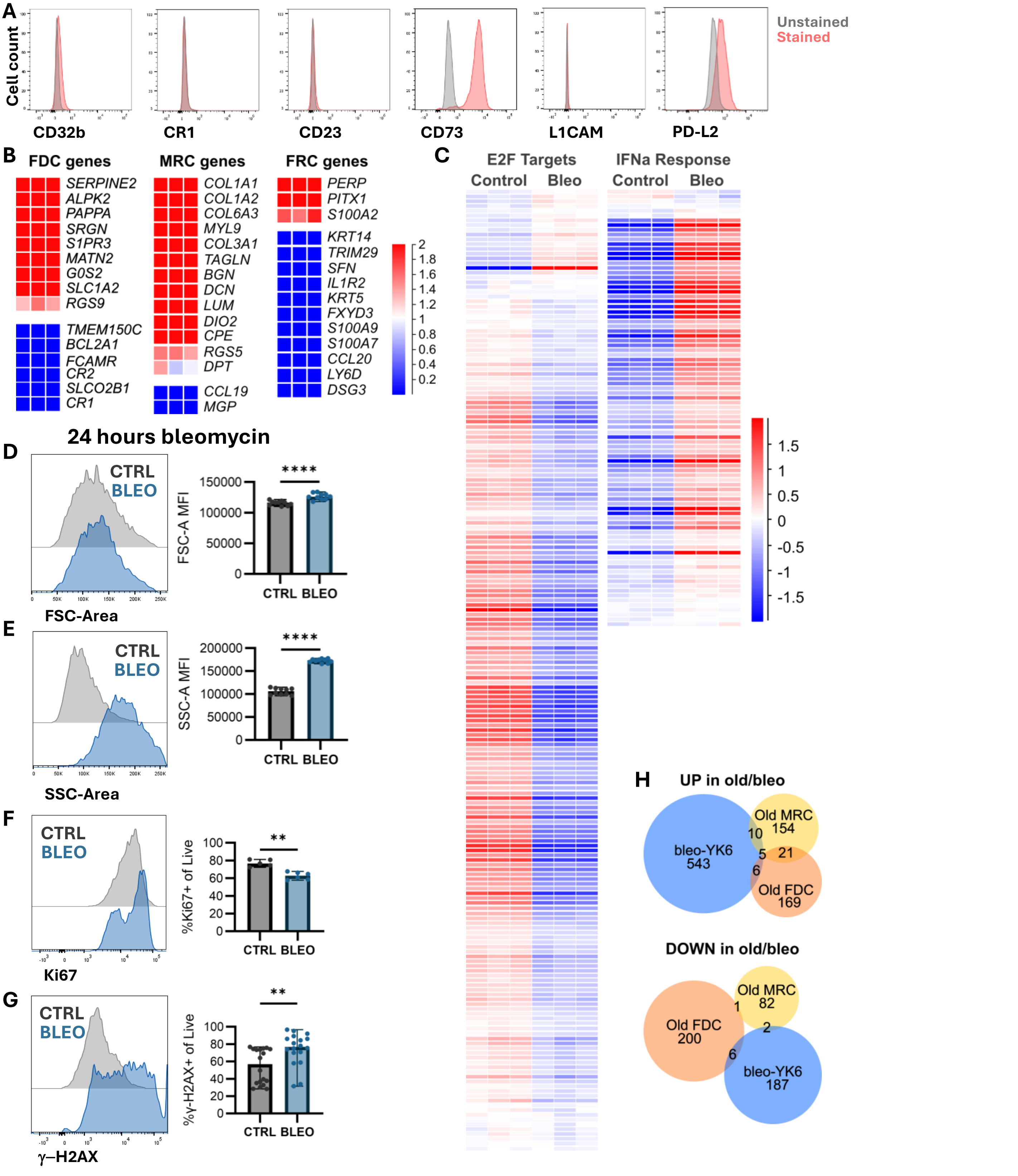


**Figure S2: Characteristics of control- and Bleo-YK6 fibroblasts.** (**A**) Expression of canonical FDC markers by YK6 cells, as determined by flow cytometry. Data are representative of at least three independent experiments. (**B**) Heatmaps showing expression of FDC, MRC and FRC genes in log2(RPM) in control YK6 fibroblasts. (**C**) Heatmaps showing expression of genes in the E2F targets (left) and IFNa response (right) pathways in control- and Bleo-YK6 fibroblasts ($log_2$(RPM); normalised within each gene). (**D-G**) Flow cytometric analysis of YK6 fibroblasts after 24hrs of bleomycin treatment. Fibroblast size (FSC, **D**) and granularity (SSC, **E**), alongside the proportion of proliferating (Ki67+, **F**) and γ-H2AX+ (**G**) cells were determined. Data are representative of five independent experiments with 5-6 replicate bleomycin treatments in each experiment. Gating strategy shown in Fig S3A. Statistical significance was determined with Mann-Whitney test, *p<0.05, **p<0.01, *** p<0.001, ****p<0.0001. (**H**) Overlaps of genes up- (top) or down- (bottom) regulated in aged MRC, FDC and Bleo-YK6 cells, as determined by DESeq2 analysis.

**A**

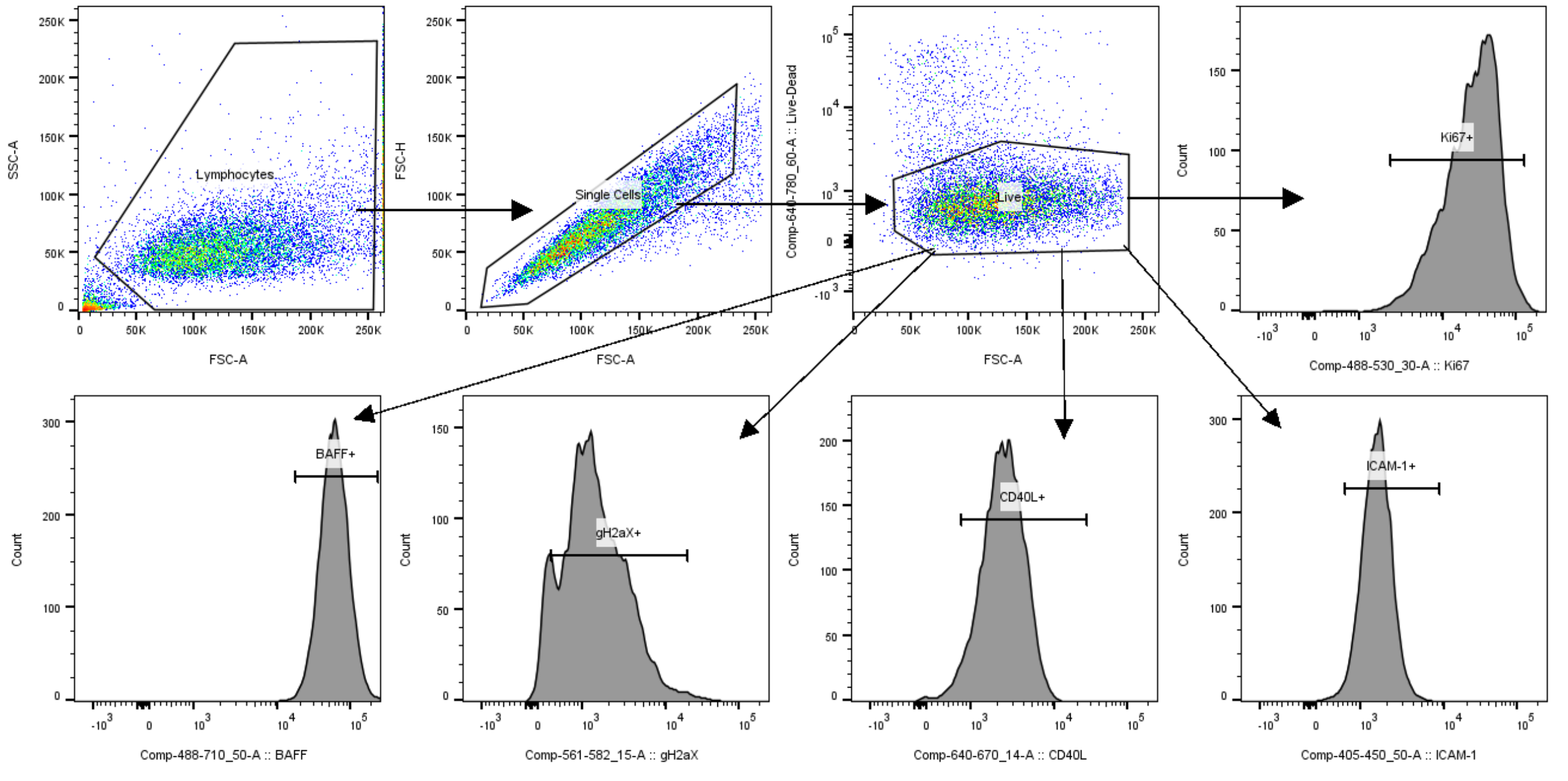


**Figure S3: Flow cytometry gating for phenotyping YK6 cells.** (**A**) Gated on total cells ('lymphocytes'), single cells and live cells. Expression of ki67, BAFF, γ-H2AX, CD40L and ICAM-1 were determined with unstained controls.

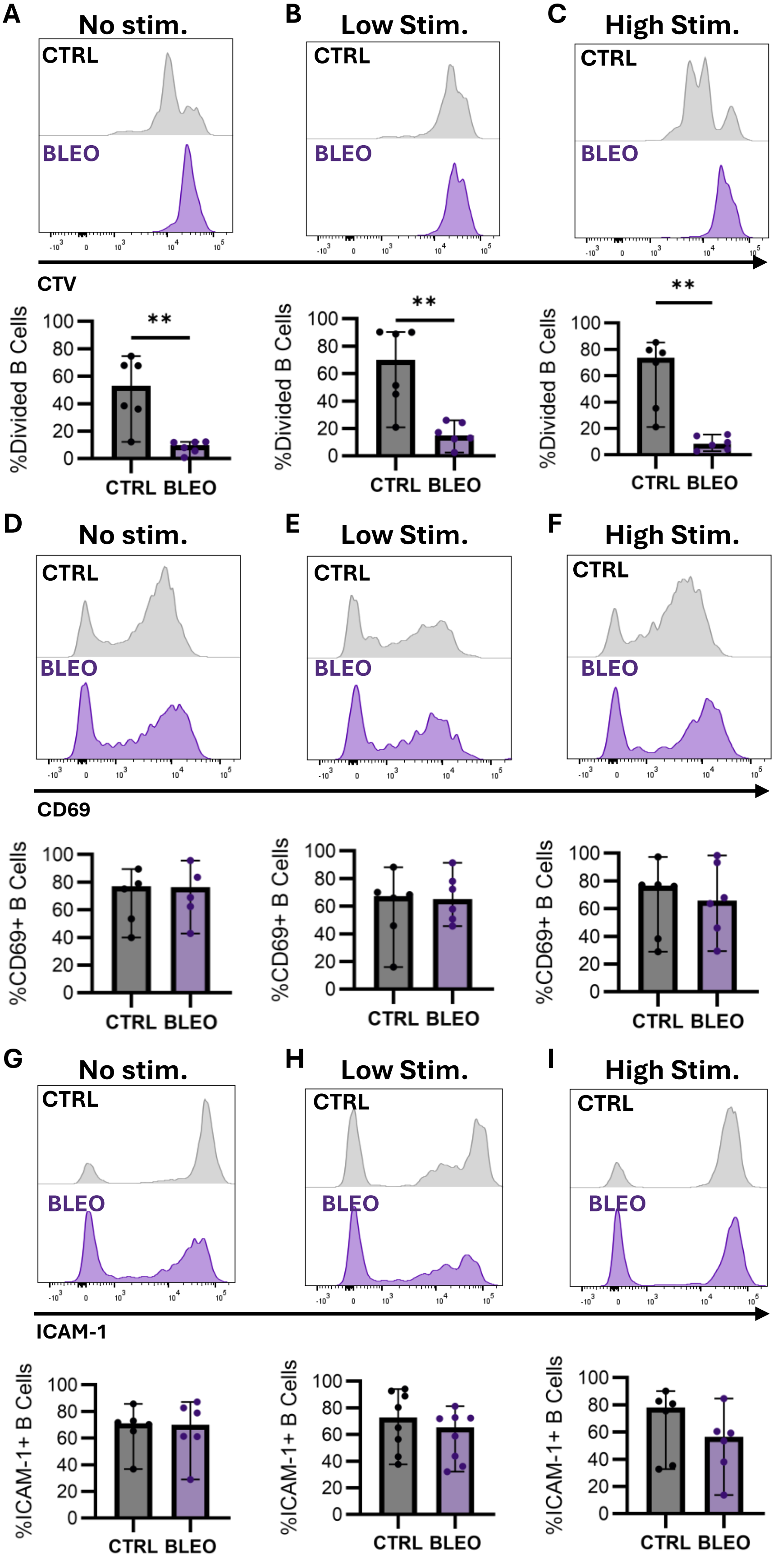


**Figure S4: Bleomycin-treated YK6 fibroblasts impair B cell proliferation, but not activation, 2D co-culture.** Naïve-enriched PBMC-derived B cells were co-cultured in 2D with control or Bleo-YK6 fibroblasts in no-, low- or high- B cell stimulation conditions and analysed by flow cytometry four days later. (**A-C**) Representative flow cytometry histograms (top) and proportion of divided B cells (bottom) for no- (**A**), low- (**B**) and high- (**C**) stimulation conditions. (**D-F**) Representative flow cytometry histograms and proportion of CD69+ B cells for no- (**D**), low- (**E**) and high- (**F**) stimulation conditions. (**G-I**) Representative flow cytometry histograms and proportion of ICAM-1+ B cells for no- (**G**), low- (**H**) and high- (**I**) stimulation conditions. Gating strategy shown in Fig S1A. Data are pooled from four independent experiments representing six PBMC donors. Statistical significance was determined with Mann-Whitney test, *p<0.05, **p<0.01, *** p<0.001, ****p<0.0001.

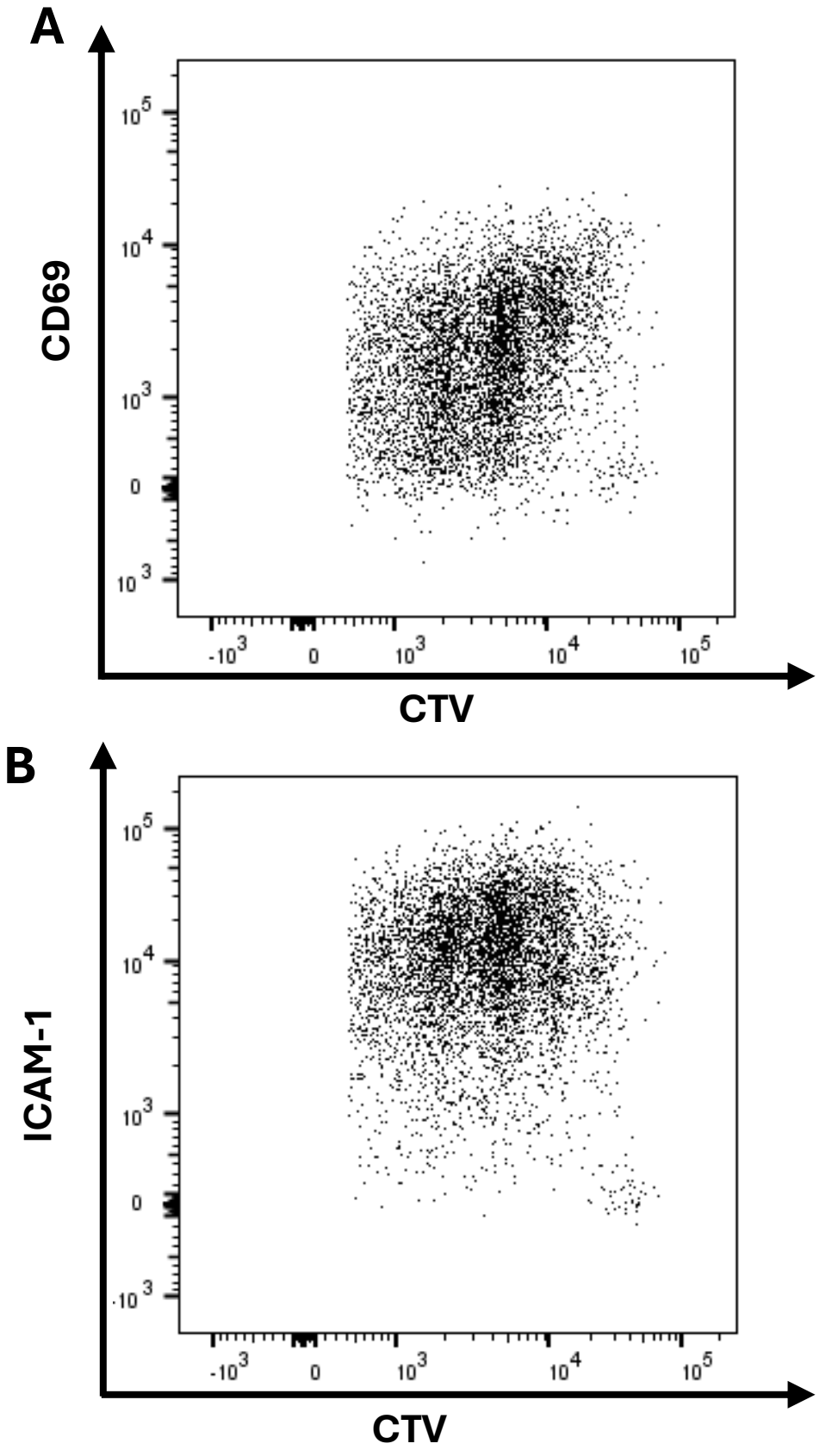



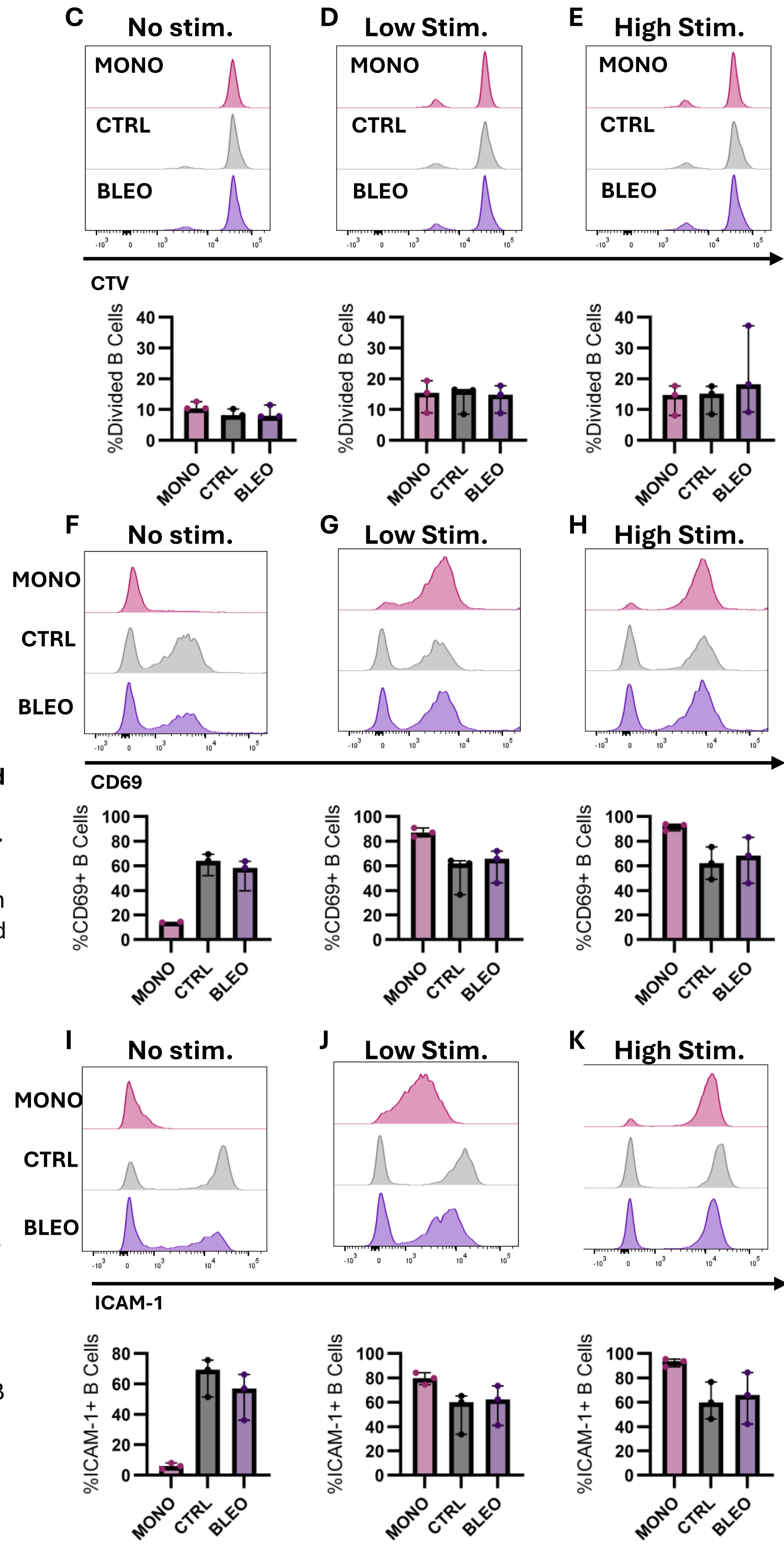



**Figure S5: B cells are equally activated in the presence of control- or bleomycin-treated YK6 fibroblasts.** (**A-B**) Representative flow cytometry dot plots showing division-linked expression of CD69 (**A**) and ICAM-1 (**B**) in stimulated B cells after four days in 3D co-culture with control YK6 fibroblasts. (**C-K**) Naïve-enriched human B cells were isolated from PBMCs and stimulated in co-culture with control- or Bleo-YK6 fibroblasts. Cells were harvested and stained for flow cytometry 48 hours later. (**C-E**) Representative flow cytometry histograms and proportion of B cells that divided for unstimulated (**C**), low-stimulated (**D**) and high-stimulated (**E**) B cells. (**F-H**) Representative flow cytometry histograms and proportion of CD69+ B cells for unstimulated (**F**), low-stimulated (**G**) and high-stimulated (**H**) B cells. (**I-K**) Representative flow cytometry histograms and proportion of ICAM-1+ B cells for unstimulated (**I**), low-stimulated (**J**) and high-stimulated (**K**) B cells. Gating strategy shown in Fig S1A. Data are pooled from two independent experiments representing three PBMC donors. Statistical significance was determined with Kruskal-Wallis test with Dunn's multiple comparisons, *p<0.05, **p<0.01, *** p<0.001, ****p<0.0001.

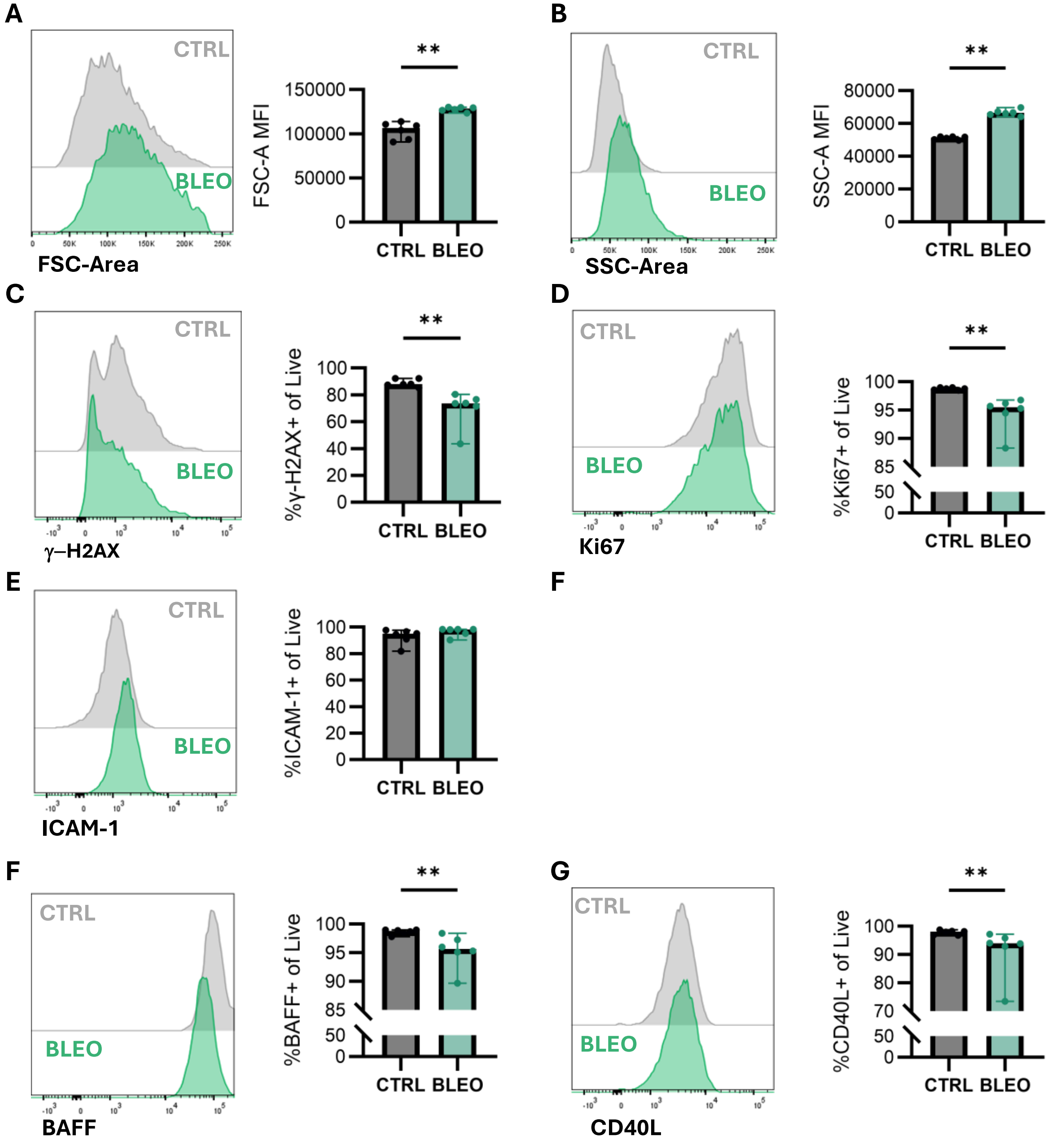


**Figure S6: Bleo-YK6 conditioned media subtly affects YK6 fibroblast phenotype**

YK6 fibroblasts were cultured in CM from control or Bleo-YK6 fibroblasts. Five days later YK6 fibroblast size (**A**) and granularity (**B**) were determined by gMFI of FSC and SSC, respectively, alongside the proportion of γ-H2AX+ (**C**), ki67+ (**D**), ICAM-1 (**E**), BAFF (**F**), and CD40L (**G**) cells. Gating strategy shown in Fig S3. Data are representative of three independent experiments with n=5-6 CM treatments per experiment. Statistical significance was determined with Mann-Whitney test, *p<0.05, **p<0.01, *** p<0.001, ****p<0.0001.

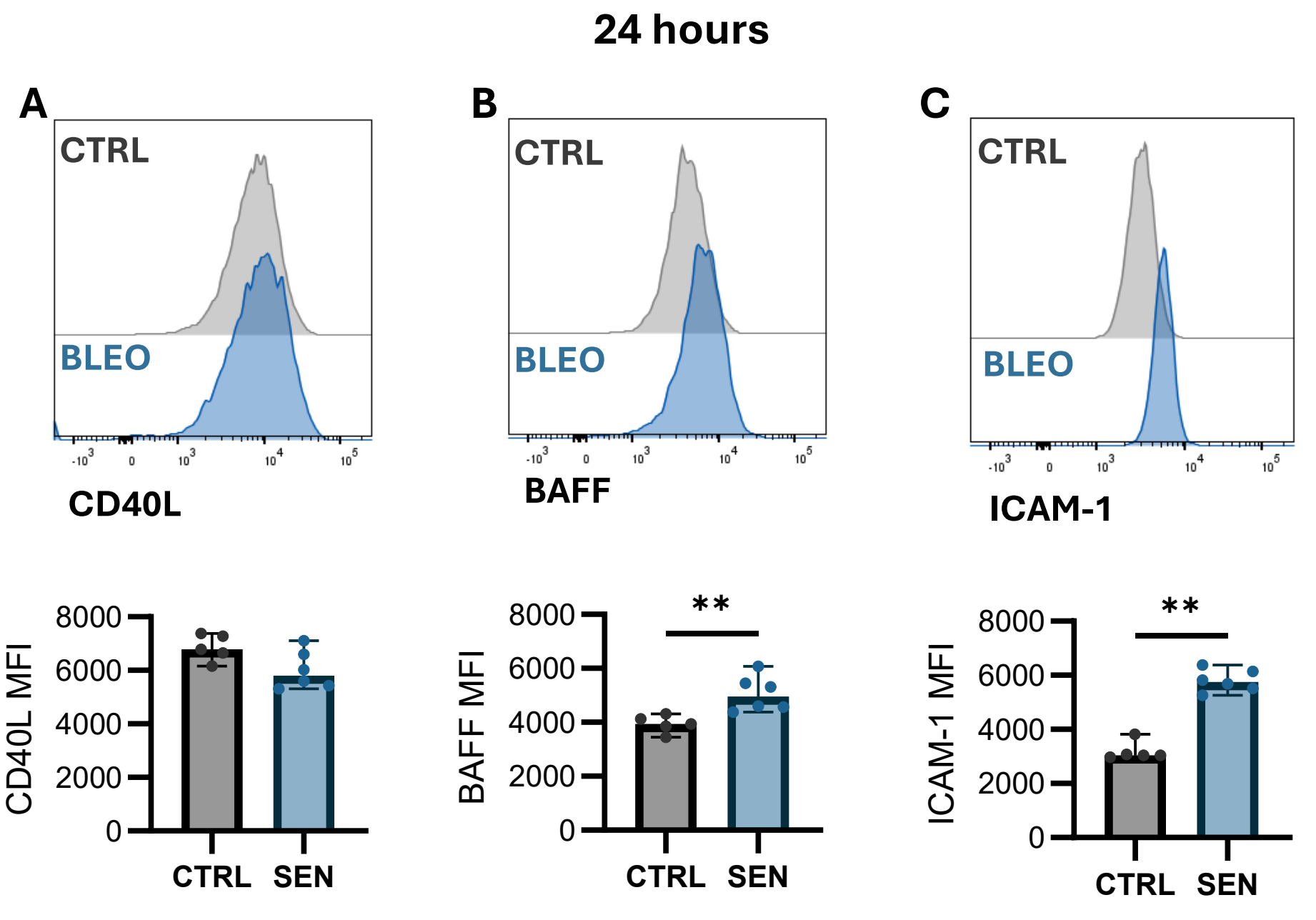


**Figure S7: 24hrs of bleomycin treatment has a minor impact on expression of ICAM and BAFF and ICAM-1 by YK6 fibroblasts.**

YK6 fibroblasts were treated with bleomycin for 24 hours and the gMFI of CD40L (**A**), BAFF (**B**), and ICAM-1 (**C**) was determined using flow cytometry. Gating strategy shown in Fig S3. Data are representative of four independent experiments with n=5-6. Data are representative of six independent experiments with n=5-8. Statistical significance was determined with Mann-Whitney test, *p<0.05, **p<0.01.
